# More than Enzymes That Make or Break Cyclic Di-GMP—Local Signaling in the Interactome of GGDEF/EAL Domain Proteins of *Escherichia coli*

**DOI:** 10.1128/mBio.01639-17

**Published:** 2017-10-10

**Authors:** Olga Sarenko, Gisela Klauck, Franziska M. Wilke, Vanessa Pfiffer, Anja M. Richter, Susanne Herbst, Volkhard Kaever, Regine Hengge

**Affiliations:** a Institut für Biologie/Mikrobiologie, Humboldt-Universität zu Berlin, Berlin, Germany; b Institut für Pharmakologie/ZFA Metabolomics, Medizinische Hochschule Hannover, Hannover, Germany; University of Chicago

**Keywords:** biofilms, c-di-GMP, cellulose, curli, diguanylate cyclase, second messenger

## Abstract

The bacterial second messenger bis-(3′-5′)-cyclic diguanosine monophosphate (c-di-GMP) ubiquitously promotes bacterial biofilm formation. Intracellular pools of c-di-GMP seem to be dynamically negotiated by diguanylate cyclases (DGCs, with GGDEF domains) and specific phosphodiesterases (PDEs, with EAL or HD-GYP domains). Most bacterial species possess multiple DGCs and PDEs, often with surprisingly distinct and specific output functions. One explanation for such specificity is “local” c-di-GMP signaling, which is believed to involve direct interactions between specific DGC/PDE pairs and c-di-GMP-binding effector/target systems. Here we present a systematic analysis of direct protein interactions among all 29 GGDEF/EAL domain proteins of *Escherichia coli*. Since the effects of interactions depend on coexpression and stoichiometries, cellular levels of all GGDEF/EAL domain proteins were also quantified and found to vary dynamically along the growth cycle. Instead of detecting specific pairs of interacting DGCs and PDEs, we discovered a tightly interconnected protein network of a specific subset or “supermodule” of DGCs and PDEs with a coregulated core of five hyperconnected hub proteins. These include the DGC/PDE proteins representing the c-di-GMP switch that turns on biofilm matrix production in *E. coli*. Mutants lacking these core hub proteins show drastic biofilm-related phenotypes but no changes in cellular c-di-GMP levels. Overall, our results provide the basis for a novel model of local c-di-GMP signaling in which a single strongly expressed master PDE, PdeH, dynamically eradicates global effects of several DGCs by strongly draining the global c-di-GMP pool and thereby restricting these DGCs to serving as local c-di-GMP sources that activate specific colocalized effector/target systems.

## IMPORTANCE

c-di-GMP signaling in bacteria is believed to occur via changes in cellular c-di-GMP levels controlled by antagonistic and potentially interacting pairs of diguanylate cyclases (DGCs) and c-di-GMP phosphodiesterases (PDEs). Our systematic analysis of protein-protein interaction patterns of all 29 GGDEF/EAL domain proteins of *E. coli*, together with our measurements of cellular c-di-GMP levels, challenges both aspects of this current concept. Knocking out distinct DGCs and PDEs has drastic effects on *E. coli* biofilm formation without changing the cellular c-di-GMP level. In addition, rather than generally coming in interacting DGC/PDE pairs, a subset of DGCs and PDEs operates as central interaction hubs in a larger "supermodule," with other DGCs and PDEs behaving as “lonely players” without contacts to other c-di-GMP-related enzymes. On the basis of these data, we propose a novel concept of “local” c-di-GMP signaling in bacteria with multiple enzymes that make or break the second messenger c-di-GMP.

## INTRODUCTION

In many bacterial species, the nucleotide second messenger bis-(3′-5′)-cyclic diguanosine monophosphate (c-di-GMP) activates genes involved in biofilm formation or directly stimulates the biosynthesis and secretion of the exopolysaccharides found in the extracellular matrix of bacterial biofilms. Cellular c-di-GMP is controlled by antagonistic enzymes, i.e., diguanylate cyclases (DGCs [with GGDEF domains]) and specific phosphodiesterases (PDEs [which can feature either EAL or HD-GYP domains]). Most bacterial species contain multiples of these enzymes as well as enzymatically inactive degenerate members of these protein families (1–4). Thus, *Escherichia coli* K-12 has 29 GGDEF/EAL domain proteins, including 12 DGCs and 13 PDEs (5, 6). Despite this multiplicity, single distinct DGCs and/or PDEs can generate distinct and highly specific outputs suggesting localized operation in conjunction with specific targets (7–9). In a few cases, it has been shown that this local c-di-GMP signaling relies on direct protein-protein interactions of a DGC and/or a PDE with a specific c-di-GMP-binding effector/target system (10–13).

Protein interactions in these complexes can play a scaffolding role; i.e., such interactions can establish a local source and sink of c-di-GMP in the immediate vicinity of a c-di-GMP-controlled molecular machinery (14). This is exemplified by the antagonistic pair DgcO/PdeO (also termed DosC/DosP); both DgcO and PdeO bind to RNase E and seem to control the equally RNase E-associated and c-di-GMP-binding PNPase within a specialized form of the degradosome of *E. coli* (15, 16). In addition (or alternatively), physical contacts among DGCs, PDEs, and target proteins can also assume a directly activating or inhibitory function (2, 14). Here, the paradigm is a c-di-GMP-mediated switch in *E. coli*, where a PDE (PdeR) and a DGC (DgcM) directly interact with each other as well as with the transcription factor MlrA. Depending on the expression and activity of yet another antagonistic DGC/PDE pair (DgcE/PdeH), c-di-GMP can accumulate and can then be bound and cleaved by the “trigger PDE” PdeR. This releases DgcM and MlrA from direct inhibition by PdeR, which allows DgcM/MlrA-driven expression of the biofilm regulator CsgD (13, 14, 17).

These examples suggested that at least some DGCs and PDEs may operate as cognate pairs of interacting proteins in specific c-di-GMP control modules that also comprise particular c-di-GMP-binding effector components and output-generating targets (2). Based on a recent analysis of the interaction of the GGDEF domain of the DGC GcbC with the degenerate EAL domain of the c-di-GMP-binding effector LapD in *Pseudomonas aeruginosa*, it was even postulated that GGDEF and EAL domains may in general interact with each other via specific amino acid patches (their α5 and α2 helices, respectively) (12). In order to test the hypothesis of the presence of specific DGC/PDE pairs, the study presented here included systematic analysis of the potential for direct interactions between all DGCs and PDEs of *E. coli*. In addition, the four proteins with degenerate GGDEF or EAL domains were included, since enzymatic activity would not seem a prerequisite for putative interactions between GGDEF and EAL domains.

Another relevant regulatory interaction is the homodimerization of GGDEF domains, which is absolutely required to activate DGCs and usually seems to depend on signal input via an N-terminal sensory domain (18, 19). However, with a total of 19 GGDEF domains in *E. coli*—present in the 12 active DGCs as well as in degenerate versions that can occur alone (CdgI) or are linked to active or inactive EAL domains—we considered and systematically tested the possibility of heterodimerization between all these GGDEF domains. Since protein-protein interactions and their potential consequences (e.g., inhibition or sequestration) depend on the cellular concentrations of the interaction partners, we also determined the cellular levels of all GGDEF/EAL domain proteins throughout all phases of the growth cycle of *E. coli*.

Taken together, our data show that c-di-GMP signaling in a bacterium with multiple DGCs and PDEs such as *E. coli* is far more complex than was anticipated. Rather than being organized in small modules of DGC/PDE pairs that act in parallel, c-di-GMP signaling seems to operate in a complex hierarchical protein interaction network with a few hubs or master controllers. These can engage in multiple contacts to more-peripheral components, with the major physiological output of this network being biofilm extracellular matrix production. In addition, there are “lonely players,” i.e., other DGCs and PDEs not engaging in mutual interactions. Overall, the protein interaction landscape of c-di-GMP signaling revealed by our study, together with our data on the cellular c-di-GMP levels in various *dgc* and *pde* mutants, led to a new model of local c-di-GMP signaling and provided the basis for precise hypotheses on the regulation and function of distinct GGDEF/EAL domain proteins in *E. coli* that will be crucial for the design of future studies.

## RESULTS

### Physical interactions between GGDEF and EAL domain proteins—does local c-di-GMP signaling operate via specific pairs of antagonistic DGCs and PDEs?

Previous studies have provided evidence that certain interacting DGC/PDE pairs can specifically affect distinct output functions (13, 17, 20). Besides interacting with each other or undergoing homomeric dimerization, the cytoplasmic GGDEF and EAL domains of these enzymes can also interact with additional cytoplasmic domains located closer to the N terminus of DGCs and PDEs (e.g., the EAL domain of PdeR was previously shown to make direct contact with the PAS domain of DgcM [13]). In order to systematically detect these kinds of interactions of GGDEF and EAL domains *in vivo*, we used a bacterial two-hybrid (2H) system, in which potentially interacting proteins or domains are fused to the N-terminal domain of the phage lambda cI repressor (cI-NTD; expressed from pBT) and to the N-terminal domain of the alpha subunit of *E. coli* RNA polymerase (RNAP) (alpha-NTD; expressed from pTRG) (21). Interactions of the hybrid proteins in cotransformants activate the yeast *His3* gene, which allows a histidine-auxotrophic *E. coli* host strain to grow on selective plates (see Materials and Methods for details).

**FIG 1:**
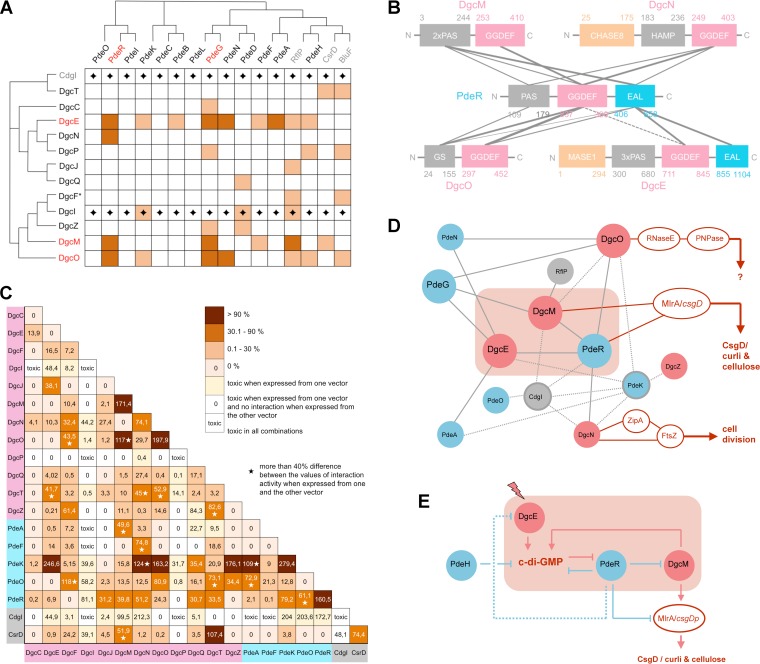
Systematic *in vivo* protein interaction patterns for all GGDEF and EAL domain proteins of *E. coli* K-12. Using the Bacterio-Match 2H system, interactions were tested for the indicated proteins and/or domains. Each protein or domain was expressed from pBT as well as from pTRG, with each interaction tested in both vector combinations (vector swap). Results were normalized for the strong interaction between RpoS (σ^S^) and RssB (23) assayed in parallel in each experiment. (A) A vector swap-integrating interaction map of (i) the 12 DGCs or the degenerate GGDEF domain protein CdgI (vertical axis) and (ii) the 13 PDEs, the degenerate EAL domain proteins RflP and BluF, or the degenerate GGDEF-EAL domain protein CsrD (horizontal axis), based on either entire proteins (if cytoplasmic) or entire cytoplasmic protein regions, which include the GGDEF and/or EAL domains. The order of proteins reflects the phylogenetic relationship between their GGDEF domains (vertical axis) and EAL domains (horizontal axis). Interactions detected in both vector combinations are indicated in dark red, and interactions found in only one vector combination are indicated in light red (for details of quantification, see Materials and Methods; separate and quantitative reciprocal vector interaction landscapes are shown in Fig. S1 and numeric data and a raw data set in Fig. S2). The five proteins exhibiting three or more interactions that were dectectable in both configurations of the vector swap (“hubs”) are highlighted in red letters and define the “core interaction module.” Data for CdgI and DgcI do not include a vector swap (indicated by diamonds), since induction of these proteins from higher-copy-number vector pTRG was toxic. DgcF (*) was obtained from *E. coli* 55989, since the *dgcF* gene is disrupted by a 5′ deletion in *E. coli* K-12 strains (6). (B) Specific domain-domain interactions between PdeR and the four DGCs found to interact with PdeR (as shown in panel A), integrating the data for the reciprocal vector combinations. Fat, thin, and dotted lines indicate different strengths of the interactions of the 2H proteins containing specific domains only (for the full quantitative data set, see Fig. S3). (C) Homomeric and heteromeric *in vivo* interactions among all 19 isolated GGDEF domains—derived from active DGCs as well as from degenerate GGDEF domain proteins—are shown as a Bacterio-Match 2H system-based interaction map. Numbers show strengths of interactions (determined as a percentage of the strength of the interaction between RpoS and RssB determined in parallel in each series of experiments) and represent averages for the respective vector swaps (an asterisk indicates a difference of >40% between the two reciprocal vector combinations). GGDEF domains with intact A-sites, i.e., those belonging to active DGCs, are highlighted in light red, degenerate GGDEF domains occurring in combination with intact EAL domains with PDE activity in light blue, and degenerate GGDEF domains not linked to an enzymatically active domain in gray. A yellow or white background color in the matrix indicates cases where induced expression of one GGDEF domain from the pTRG higher-copy-number vector resulted in toxicity; i.e., the result given is that of a single vector configuration. (D) Graphical summary of the protein-protein interaction network of GGDEF/EAL domain proteins. Lines (edges) represent direct interactions between connected proteins (only the interactions detectable in both reciprocal vector configurations of the 2H assays shown in panel A are taken into account). Gray lines represent results based on the data shown in panel A; dotted lines represent interactions between isolated GGDEF domains as shown in panel C; red lines represent direct interactions with the indicated effector/target systems previously reported and cited in the Discussion. DGCs, PDEs, and degenerate GGDEF/EAL domain proteins are shown as red, blue, and gray spheres, respectively, with a gray circle indicating interaction via a degenerate GGDEF domain. The light red box highlights the three members of the matrix control switch module (DgcE, DgcM, and PdeR) (13, 14) which, together with DgcO and PdeG, form the “core interaction hub,” i.e., the set of five interconnected proteins with more than three reciprocally detectable interactions each (highlighted in red letters in panel A). (E) The functional network of the matrix control switch module. The three proteins representing the matrix control switch module are included in the light red box as described for panel D, but instead of indicating protein-protein interactions, the lines indicate functional interactions. These can have positive/negative functional consequences (shown by arrows/blocking lines) such as either production/degradation of c-di-GMP by the indicated DGCs/PDEs or functional activation/inhibition by direct protein contacts.

In a first series of experiments, full-size DGCs and PDEs (if entirely cytoplasmic) or the cytoplasmic regions of N-terminally membrane-attached DGCs and PDEs were expressed from the two 2H vectors (including vector swaps). Putative interactions of all DGCs and the degenerate GGDEF domain protein CdgI with all PDEs and proteins carrying degenerate EAL domains (RflP, BluF, CsrA) were assayed and normalized using the well-studied interaction between *E. coli* stationary-phase sigma factor RpoS (σ^S^) and RssB, a RpoS-binding proteolytic targeting factor (22, 23), as a reference. An integrated visual summary of the data is shown in Fig. 1A, whereas the two vector-reciprocal interaction landscapes are presented separately in Fig. S1 in the supplemental material. As an exemplary raw data set (before and after normalization with the RpoS/RssB data), the data for PdeR in combination with all DGCs and CdgI are shown (Fig. S2A; similar data sets were obtained for all other proteins). The numeric data for all the combinations used for visual representations (Fig. 1 and Fig. S1) are shown in Fig. S2. Two GGDEF domain proteins, DgcI and CdgI, could be cloned but turned out to be toxic in several combinations under conditions of expression from pTRG (which has a higher copy number than pBT). This is consistent with an earlier report indicating that their genes (previously *yliF* and *yeaI*, respectively) show very low if any expression at all under standard conditions (24).

10.1128/mBio.01639-17.1 FIG S1  The 2H interaction landscape of all 29 GGDEF/EAL domain proteins of *E. coli* K-12. The Bacterio-Match 2H system interaction data correspond to those shown and described in the legend to Fig. 1, but the data for the reciprocal vector configurations are shown separately here; i.e., PDEs and degenerate EAL domain proteins were expressed from pBT and DGCs and degenerate GGDEF domain proteins from pTRG in panel A and vice versa in panel B. Data were normalized for the strong interaction between RpoS and RssB determined in parallel in each experiment and were visualized graphically using the MatLab program (numerical data are presented in Fig. S2). Download FIG S1, TIF file, 3.9 MB. Copyright © 2017 Sarenko et al. 2017 Sarenko et al. This content is distributed under the terms of the Creative Commons Attribution 4.0 International license.

10.1128/mBio.01639-17.2 FIG S2  Graphical and numerical data sets of 2H interaction experiments with all 29 GGDEF/EAL domain proteins of *E. coli* K-12. Exemplary Bacterio-Match 2H system interaction data for PdeR combined with all DGCs and the degenerate GGDEF domain protein CdgI are shown as raw data (in CFU counts) (A) and after normalization for the strong interaction between RpoS and RssB determined in parallel (B). Corresponding data sets were generated for each of the 29 GGDEF/EAL domain proteins (data not shown) and are summarized in numerical form (after normalization for RpoS/RssB interaction) for the reciprocal vector configurations as described for panels C and D. Data for DgcI and CdgI are missing in panel C because overproduction of these proteins from pTRG, which has a higher copy number than pBT, was toxic. Download FIG S2, TIF file, 4.5 MB. Copyright © 2017 Sarenko et al. 2017 Sarenko et al. This content is distributed under the terms of the Creative Commons Attribution 4.0 International license.

A principal result from this 2H analysis was the finding that DGCs and PDEs do not come in specific interaction pairs. Rather, a few DGCs and PDEs show multiple interactions in a tightly interconnected network, whereas others do not seem to make significant contacts. The small set of promiscuously interacting proteins, or “interaction hubs” (defined by three or more reciprocally demonstrated interactions), consisted of DgcE, DgcM, and DgcO as well as PdeR and PdeG (highlighted in red in Fig. 1A). The proteins shown in Fig. 1A were ordered according to the phylogenetic relationships of their GGDEF or EAL domains (with phylogenetic trees indicated along the axes of the figure). The patchy pattern of the interaction matrix does not show significant correlation with these phylogenetic relationships, suggesting that the interactions evolved independently and/or also involve specific domains besides the GGDEF and EAL domains.

The second striking result was that these five hub proteins not only have multiple interactions but together form an interconnected network that includes precisely the three components of the central switch device, i.e., DgcE, PdeR, and DgcM, which turns on the expression of the key biofilm regulator CsgD and thereby biofilm matrix production. While local signaling based on PdeR/DgcM interaction has been demonstrated previously (13), our data obtained here also suggest that DgcE has a more local and direct interaction-based function in this switch mechanism beyond just increasing the cellular c-di-GMP level. In addition, the ability of DgcE and DgcM to interact with several PDEs as well as the ability of PdeR to interact not only with DgcE and DgcM but also with DgcN and DgcO raises the possibility that these additional interaction partners may conditionally modulate the outcome of the CsgD/matrix switch.

PdeR, which has a PAS-GGDEF^deg^-EAL domain architecture, is the central decision-making component of this molecular switch (14). As an interaction hub (Fig. 1A), PdeR is able to make contacts with four different DGCs (Fig. 1A), and all three PdeR domains are involved in its interaction with DgcM (13). In order to systematically study the role of all domains in this interaction network around PdeR, the isolated domains of PdeR and the additional three interacting DGCs (DgcE, DgcN, and DgcO) were tested in the 2H assay (including vector swaps; Fig. 1B) (quantitative data sets are presented in Fig. S3). The results show that GGDEF and EAL domains play a dominant role in these interactions, with GGDEF^PdeR^ most strongly interacting with the GGDEF domains of the DGCs, whereas the EAL^PdeR^ domain showed more promiscuous contacts to GGDEF and EAL or additional N-terminal domains.

10.1128/mBio.01639-17.3 FIG S3  Complete 2H interaction data set for single domains of PdeR with single domains of DgcO, DgcN, and DgcE. The indicated Bacterio-Match 2H system interaction data are shown before (A, C, and E) and after (B, D, and F) normalization for RpoS/RssB interaction. The fully membrane-integrated N-terminal domains of DgcN and DgcE were not included in the analysis. A similar data set corresponding to the interaction between isolated domains of PdeR and those of DgcM was previously reported (13) using the old designations YciR and YdaM, respectively. Download FIG S3, TIF file, 6.6 MB. Copyright © 2017 Sarenko et al. 2017 Sarenko et al. This content is distributed under the terms of the Creative Commons Attribution 4.0 International license.

### Homomeric and heteromeric interactions between all GGDEF domains of *E. coli.*

GGDEF domains homodimerize, which is essential for DGC activity, since each monomer within the dimer binds one of the two GTP molecules that have to be linked to generate c-di-GMP (25–27). Our observation that GGDEF^PdeR^ can undergo interactions with other GGDEF domains (Fig. 1B and Fig. S3) raised the possibility that heteromeric interactions among GGDEF domains of different DGCs or proteins with degenerate GGDEF domains may be more common and could perhaps play a regulatory role. In order to systematically analyze such interactions, we generated a full 2H interaction matrix of all 19 isolated GGDEF domains (i.e., 12 from the active DGCs and the 7 degenerate GGDEF domains present in active PDEs or in enzymatically inactive proteins), with each expressed in both vectors (results from the vector swaps were averaged, which resulted in the half-panel of the 19-by-19 combinations shown in Fig. 1C; note that induced expression of some isolated GGDEF domains from the higher-copy-number plasmid pTRG was toxic; i.e., in these cases, results are from one vector configuration only).

Overall, not only did we observe a distinct set of highly specific homo- and heteromeric interactions, but some of these interactions were the strongest that we have ever observed in the Bacterio-Match 2H system, with some being more than 2-fold stronger than the tight interaction between RpoS and RssB. A homomeric dimerization of isolated GGDEF domains of the 12 active DGCs was observed in only two cases, i.e., for GGDEF^DgcM^ and GGDEF^DgcO^. Among the seven degenerate GGDEF domains, only GGDEF^PdeK^ and GGDEF^PdeR^ showed strong homomeric dimerization.

With respect to heteromeric GGDEF-GGDEF contacts, GGDEF^PdeK^ and GGDEF^CdgI^ showed multiple specific and strong interactions with several other GGDEF domains. CdgI is a degenerate GGDEF domain protein of unknown function which shares an N-terminal domain with the active DGCs DgcT and DgcX (the latter is an extra DGC mainly found in enteroaggregative *E. coli*) (6). While CdgI has a degenerate A-site (the GGDEF motif corresponding to the active center of DGCs), it exhibits an intact I-site (a secondary c-di-GMP binding site involved in allosteric control of many active DGCs [26]) (Fig. S4A). We observed that purified CdgI could indeed bind c-di-GMP (Fig. S4B). Finally, the degenerate GGDEF domain of CsrD showed a single direct and strong interaction with another GGDEF domain, i.e., the one in DgcT.

10.1128/mBio.01639-17.4 FIG S4  CdgI is a c-di-GMP-binding protein. CdgI consists of a fully membrane-inserted N-terminal MASE4 domain linked to a GGDEF domain with an intact I-site but a degenerate A-site (A). The same MASE4-GGDEF domain architecture is also found in two active DGCs of *E. coli*, DgcT and DgcX, with the latter not occurring in *E. coli* K-12 but occurring in enteroaggregative *E. coli*. The isolated CdgI GGDEF domain was purified, and binding of radiolabeled c-di-GMP or GTP was assayed by UV cross-linking (B), with nonradiolabeled c-di-GMP added at the indicated concentrations. No binding of radiolabeled GTP (at the same concentration as the radiolabeled c-di-GMP) was observed (last lane). Download FIG S4, TIF file, 9.8 MB. Copyright © 2017 Sarenko et al. 2017 Sarenko et al. This content is distributed under the terms of the Creative Commons Attribution 4.0 International license.

### Cellular levels and physiological regulatory patterns of GGDEF/EAL domain proteins in *E. coli.*

**FIG 2:**
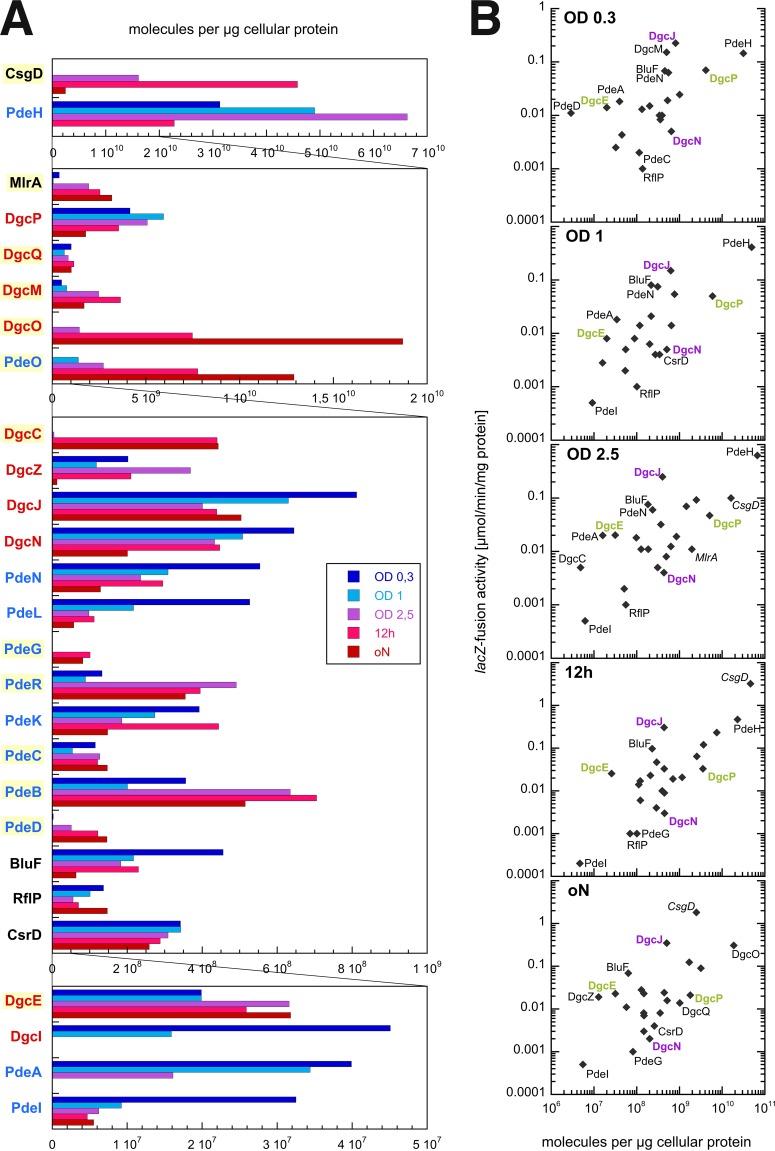
Cellular levels and expression patterns of all GGDEF/EAL domain proteins of *E. coli* K-12. (A) Derivatives of strain W3110 expressing chromosomally encoded C-terminally 3× FLAG-tagged versions of GGDEF and/or EAL proteins as indicated were grown in LB medium at 28°C. Samples were taken at five representative stages during the growth curve (i.e., at optical densities at 578 [OD_578_] of 0.3, 1, and 2.5 and after 12 h of incubation [12h] and overnight incubation [oN]). Levels of specific proteins were determined by immunoblot analysis (using antibodies against 3× FLAG tag) with dilutions of purified PdeL::3× FLAG on the same SDS gels used for normalization (see Fig. S5). Data shown here represent averages of results from two biological replicates. A linear scale for protein abundance is used as this allows better visualization of variations in the levels of individual proteins along the growth cycle. However, since the variations in protein abundance among all detectable GGDEF/EAL domain proteins extended over more than 3 orders of magnitude, different scales had to be used for different sets of proteins, with panels with successively smaller scales ordered from top to bottom. Based on average cellular-protein-to-cell-number ratios for *E. coli* (58, 63), 10^7^ molecules per μg cellular protein corresponds to approximately 1 molecule per cell, which—given the dimensions of *E. coli* K-12 cells in the postexponential growth phase (63)—corresponds to approximately 1 nM. Among the 29 GGDEF/EAL domain proteins, only four (DgcI, DgcF, DcgT, and CdgI) were not detectable. Proteins known to be under RpoS control are highlighted in yellow. (B) For comparisons of the relative levels of transcriptional activity and cellular protein, the specific activities of respective single-copy *lacZ* reporter fusions measured under the same conditions and previously published (24) were plotted against the protein level data as shown in panel A. Examples of proteins showing strong differences in gene expression but similar protein levels and vice versa are highlighted in purple and green, respectively.

Interaction of certain proteins can potentially play a physiological role only if these proteins have a chance to “see” each other, i.e., if they are expressed under the same physiological conditions. In addition, a significant functional consequence of direct protein interactions—whether in the form of activation, inhibition, or sequestration—depends on adequate stoichiometries of the binding partners. In order to systematically measure cellular levels of all GGDEF/EAL domain proteins, we inserted a FLAG-tag-encoding sequence at the 3′ end of all corresponding genes in the chromosome. All proteins were quantified by immunoblot analysis along the entire growth cycle of these strains in complex medium (Fig. 2A and Fig. S5).

10.1128/mBio.01639-17.5 FIG S5  Complete immunoblot data set for the determination of cellular levels of detectable GGDEF/EAL domain proteins. Derivatives of strain W3110 carrying 3× FLAG-tagged fusions at the 3′ ends of the GGDEF/EAL genes as indicated in the respective rows were grown in LB at 28°C. At OD_578_ levels of 0.3, 1, and 2.5 as well as after 12 h of incubation and overnight (oN) incubation, samples were taken for immunoblot analysis (using antibodies against the 3× FLAG tag). Each row shows a representative image of cellular 3× FLAG-tagged protein levels (left panel) and the respective samples of purified C-terminally 3× FLAG-tagged PdeL applied in different amounts (ranging between 4.48 × 10^11^ molecules in PdeL [see lane 1] and 1,79 × 10^8^ molecules in PdeL [see lane 10]) as the standard protein for quantification on the same gel (right panel). Although PdeL reference samples were always run on the same gel side by side with the cellular samples, images of these reference samples were cut and ordered with respect to the ranges of their concentrations (which were chosen differently due to the large differences in the cellular levels of the GGDEF/EAL domain proteins; i.e., the amount of total cellular protein loaded ranged from 1.5 to 80 µg). Each experiment was done in at least two biological replicates. The asterisk (*) indicates unspecific bands, which also appeared in the control samples from unflagged W3110 on the same immunoblot. Download FIG S5, TIF file, 5.4 MB. Copyright © 2017 Sarenko et al. 2017 Sarenko et al. This content is distributed under the terms of the Creative Commons Attribution 4.0 International license.

Of the 29 GGDEF/EAL domain proteins of *E. coli* K-12, 25 were found to be expressed. Only four proteins remained at levels below detection: DgcT and CdgI, which are known to be subject to CsrA repression (28); DgcF, which is not expressed due to a deletion that includes the 5′ end of its gene specifically in *E. coli* K-12 (6); and PdeF, which is induced anaerobically (29). For the 25 quantifiable proteins, expression levels differed by more than 3 orders of magnitude (Fig. 2A). The protein that was most strongly expressed by far was PdeH, which accumulated to >5.000 molecules/cell during the postexponential growth phase. In the stationary phase, however, it nearly disappeared. This is consistent with *pdeH* being a σ^FliA^-dependent flagellar class 3 gene (30) and flagellar genes being switched off during the transition into stationary phase (31). Relatively strongly expressed DGCs present during all phases of growth were DgcP and DgcQ, but during the stationary phase, DgcM and, in particular, DgcO took over as the most highly expressed DGCs (Fig. 2A).

Induction during the stationary phase was observed for a number of DGCs and PDEs—including DgcM, DgcO, PdeO (the latter two of which are encoded in an operon), DgcC, PdeG, PdeR, PdeB, and PdeD—and corresponds to the previously reported RpoS regulation of their genes (24). In contrast, proteins with vegetative control of expression (mediated by RpoD-RNAP; e.g., DgcP, DgcZ, DgcN, PdeN, PdeL, and BluF) in general showed reduced levels in the stationary phase. Taken together, these specific expression patterns indicate that the complements of DGCs and PDEs are remarkably different in growing and stationary-phase cells. In particular, the DGCs and PDEs that represent the core network of interaction hubs (Fig. 1A), i.e., DgcE, DgcM, PdeR, DgcO, and PdeG, are all induced in the stationary phase (for DgcE, see also below), which corresponds to the previously reported finding that *lacZ* reporter fusions to their genes are under the control of RpoS (24).

GGDEF/EAL domain protein levels as determined here were also compared with the levels of expression of respective single-copy *lacZ* fusions, which essentially reflect transcriptional activities of the corresponding genes as reported earlier (24). Despite the expected broad correlation (Fig. 2B), protein levels were found to vary by up to 2 orders of magnitude for similarly expressed genes and vice versa (compare, e.g., DgcE/DgcP and DgcJ/DgcN [highlighted in Fig. 2B]). While this can reflect very different translational efficiencies and posttranscriptional control at the RNA level (e.g., by CsrA [28] or small RNAs [32]), unexpectedly low protein levels could also be due to proteolysis. DgcE and PdeA indeed showed a series of smaller proteolytic fragments in the immunoblot analyses (Fig. S6), with the sizes of these fragments indicating cleavage between domains or processive proteolysis from the N terminus with pausing at domain boundaries (note that the tag for visualization is located at the C terminus). When these shorter fragments were taken into account for the quantification (Fig. S6), DgcE showed stationary-phase induction as expected from earlier reports on *dgcE* gene regulation (17, 24).

**FIG 3:**
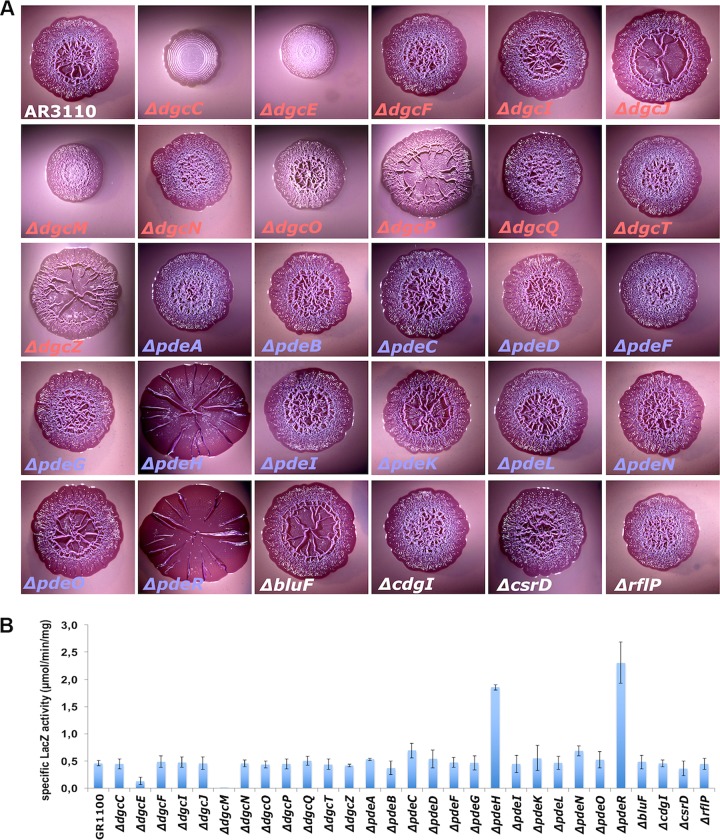
Effects of knockout mutations in all GGDEF/EAL genes of *E. coli* K-12 on biofilm matrix as detected by macrocolony morphology and the expression of curli structural genes. (A) Macrocolony morphology of curli fibers and cellulose-producing strain AR3110 and the indicated mutant derivatives after growth on CR plates at 28°C for 5 days. Buckling of macrocolonies to form ridges, rings, and wrinkles depends on the amounts, on assembly into a nanocomposite, and on the spatial distribution of curli fibers and cellulose. For more details, see the text. (B) Expression of a single-copy *csgB*::*lacZ* reporter fusion. Derivatives of strain W3110 *Δlac*(*I-A*) carrying *csgB*::*lacZ* and deletion mutations in the indicated GGDEF/EAL domain-encoding genes were grown in liquid LB medium at 28°C. Specific β-galactosidase activities were determined in overnight cultures.

10.1128/mBio.01639-17.6 FIG S6  A role for proteolysis in the control of PdeA and DgcE. For PdeA and DgcE expressed as C-terminally 3× FLAG-tagged proteins from their chromosomal genes, ladders of degradative fragments instead of single protein bands were detected in the immunoblot analysis (for details, see the Fig. 3 legend). The largest visible bands correspond to the full-size proteins. Also taking into account the proteolytic fragments for the quantification changed the expression pattern (“DgcE” to “DgcE all,” “PdeA” to “PdeA all”), such that the pattern reflects RpoS control and induction during entry into the stationary phase for DgcE, whereas PdeA is vegetatively controlled (by the vegetative sigma factor RpoD) (24), with its cellular level decreasing during entry into the stationary phase. Download FIG S6, TIF file, 5.9 MB. Copyright © 2017 Sarenko et al. 2017 Sarenko et al. This content is distributed under the terms of the Creative Commons Attribution 4.0 International license.

### A core set of DGCs and PDEs determines biofilm matrix production without affecting the cellular c-di-GMP level.

Our data indicate that 25/29 GGDEF/EAL domain proteins are present in cells entering the stationary phase, when the c-di-GMP/RpoS-dependent biofilm regulator CsgD is induced and therefore amyloid curli fibers and cellulose are produced. In order to see which of these GGDEF/EAL domain proteins contribute significantly to CsgD and matrix production, all the corresponding 29 knockout derivatives of *E. coli* K-12 strain AR3110, which produces both matrix components (33), were tested for their macrocolony morphology (Fig. 3), which reflects curli and cellulose production. Only five mutants exhibited a strong phenotype in this assay (Fig. 3A). *ΔdgcE* and *ΔdgcM* mutations strongly reduced overall wrinkling (and also yielded smaller but thicker colonies), which reflects reduced levels of curli and cellulose. The *ΔdgcC* mutation resulted in a concentric ring morphology indicative of impaired cellulose biosynthesis but normal curli production (33, 34). Knocking out PdeH or PdeR resulted in enlarged, extremely flat and stiff macrocolonies that only occasionally buckled into long radial ridges, i.e., showed a phenotype reflecting increased production of both curli and cellulose (33, 34). When curli gene expression was assayed in liquid growth medium by using a single-copy *csgB*::*lacZ* reporter fusion, a similar result was obtained (Fig. 3B); i.e., the *ΔdgcE* and *ΔdgcM* mutations strongly reduced expression of *csgB*::*lacZ*, while the *ΔpdeH* and *ΔpdeR* mutations enhanced expression. The *ΔdgcC* mutation did not affect the expression of *csgB*::*lacZ*, which confirmed that DgcC acts specifically on cellulose biosynthesis. The other DGCs and PDEs were present but did not contribute to the control of matrix production under our standard growth conditions.

**FIG 4:**
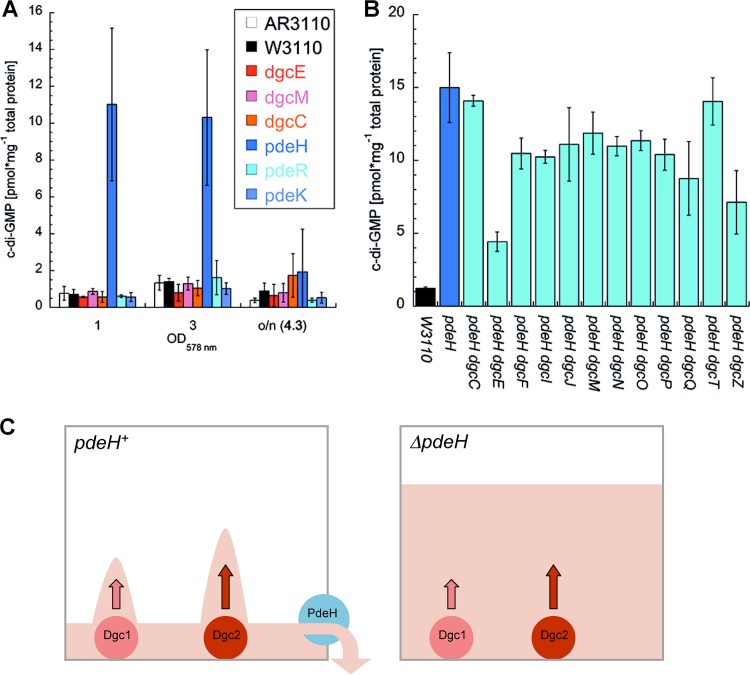
Cellular levels of c-di-GMP and a novel model of local c-di-GMP signaling. (A) Cellular c-di-GMP concentrations were determined for strains AR3110 and W3110 and for strain W3110 carrying mutations that affect biofilm matrix production (as shown in this panel) at different stages of the growth cycle (growing at 28°C in LB medium and sampled at the indicated OD_578_ levels and overnight). (B) Cellular c-di-GMP concentrations were determined for derivatives of the W3110 *pdeH* mutant also carrying secondary mutations that eliminate the 12 DGCs of *E. coli* K-12 (grown as described for panel B, with samples taken at an OD_578_ of 3). One picomole/mg cellular protein corresponds to approximately 60 molecules per cell or a cellular concentration of 60 nM (see also Fig. 2 legend). (C) The “fountain model” of local c-di-GMP signaling. In wild-type cells, the strongly expressed PdeH acts as a drain to maintain a low level of the cellular pool of c-di-GMP, with localized production by certain DGCs (the “fountains”) allowing activation of directly associated effector/target systems. In the absence of PdeH as a drain, local signaling is lost since the activities of the producing DGCs combine to drive up the level of the global cellular c-di-GMP pool.

Cellular c-di-GMP concentrations were determined for mutants that showed clear-cut effects on curli and cellulose production (Fig. 4A). In the presence of the full complement of GGDEF/EAL domain proteins, the c-di-GMP level was 0.7 pmol/mg total cellular protein at an optical density at 578 nm (OD_578_) of 1, which was transiently raised 2-fold during the transition into stationary phase (at an OD_578_ of 3). This corresponds to surprisingly low cellular c-di-GMP concentrations of approximately 40 and 80 nM, respectively. In contrast, the *ΔpdeH* mutant reached >10 pmol/mg total cellular protein (corresponding to a cellular c-di-GMP concentration of approximately 0.6 μM). This indicates that under these conditions, which coincide with its major expression, PdeH is the major PDE affecting the overall cellular c-di-GMP pool. In contrast, the *ΔdgcE*, *ΔdgcM*, *ΔdgcC*, and *ΔpdeR* mutations—despite their equally pronounced effects on macrocolony morphology and curli and/or cellulose production—did not significantly affect the cellular c-di-GMP level in otherwise wild-type backgrounds. This is a clear indication that these three DGCs as well as PdeR act in a local manner instead of controlling the cellular c-di-GMP level.

In order to find out which DGC was responsible for the drastically increased cellular c-di-GMP level in the *ΔpdeH* mutant, we knocked out all *dgc* genes in the *ΔpdeH* background. Eliminating several DGCs reduced c-di-GMP levels by 20% to 50% (Fig. 4B), indicating that these proteins are indeed active DGCs. However, these secondary *dgc* mutations did not affect macrocolony morphology (Fig. S7). In contrast, eliminating DgcC or DgcM had the opposite consequences, i.e., no or little reduction of the very high c-di-GMP level (Fig. 4B) but drastic changes in matrix production, with the macrocolony morphology being similar that that seen with the *ΔdgcC* and *ΔdgcM* single mutants with intact PdeH (compare Fig. S7 and Fig. 3). Finally, knocking out *dgcE* in the *ΔpdeH* background brought down the cellular c-di-GMP level by about 75%; i.e., DgcE was clearly the major DGC responsible for the control of the cellular c-di-GMP level under these conditions. However, the c-di-GMP level in the *ΔpdeH ΔdgcE* double mutant was still 3-fold higher than that in the parental strain (Fig. 4A)—yet, nevertheless, its macrocolony morphology (Fig. S7) indicated strong reductions of curli and cellulose levels similar to those seen with the *ΔdgcE* single mutant (Fig. 3). That DgcE contributes most to the strongly elevated c-di-GMP level in the *pdeH* mutant also explains why the *ΔdgcE* mutation strongly suppresses the nonmotile phenotype of a *pdeH* mutant as previously reported (17, 30, 35).

10.1128/mBio.01639-17.7 FIG S7  Effects on macrocolony morphology of secondary mutations in DGC genes introduced into *pdeH* mutants. Macrocolonies of the indicated strains were grown on CR plates at 28°C for 5 days. W3110 derivatives carry the *bcsQ*^stop^ mutation present in K-12 strains, which is polar with respect to the expression of *bcsAB* and thus eliminates cellulose production. AR3110 and its derivatives carry a wild-type *bcsQ* allele and therefore have intact cellulose synthase (33). Download FIG S7, TIF file, 9 MB. Copyright © 2017 Sarenko et al. 2017 Sarenko et al. This content is distributed under the terms of the Creative Commons Attribution 4.0 International license.

Overall, we conclude that, for the key components of the CsgD/matrix-controlling c-di-GMP switch—DgcE, DgcM, and PdeR—the effects on matrix production do not correlate with the effects on the cellular c-di-GMP level; i.e., this switch is the core of a local c-di-GMP signaling device that finds its correspondence in the core of the protein interaction network observed for precisely these GGDEF/EAL domain proteins (Fig. 1). Moreover, DgcC shows a similar lack of correspondence between effects on c-di-GMP levels and on cellulose production, indicating local c-di-GMP signaling by DgcC specifically in the control of cellulose synthase.

## DISCUSSION

Based on *in vivo* two-hybrid (2H) analysis, the study presented here produced a large data set corresponding to possible protein-protein and domain-domain interactions among the 29 GGDEF/EAL domain proteins of *E. coli* K-12. 2H system-based approaches are known to generate certain fractions of false positives. On the other hand, they also miss many relevant interactions and may even systematically underestimate numbers of “real” interactions (36, 37). Apparent false negatives, which can also be observed in vector swaps, can arise, for instance, by steric hindrance within the usually large complexes that must bring together at least four domains to activate the output reaction. Therefore, the goal of a two-hybrid-based interactome analysis is not to focus on individual interactions (although these may inspire deeper studies of particularly interesting single cases) but is rather to detect relevant patterns of interactions in the entire data set. Such patterns are highly likely to be biologically meaningful when they correlate with common functional patterns and coregulation of the proteins involved. Therefore, our study results combine 2H data-based interactome patterns of all GGDEF/EAL domain proteins in *E. coli* K-12 with functional data on their roles in biofilm formation and data on their control of expression during the entire growth cycle.

### High-specificity local c-di-GMP signaling in a supermodule network of interacting DGCs and PDEs switches on biofilm matrix production in *E. coli.*

As demonstrated here for *E. coli*, c-di-GMP signaling in a bacterium with multiple DGCs and PDEs is far more complex than previously anticipated. The action of all of these antagonistic enzymes does not simply sum up and converge to determine the global cellular c-di-GMP level that then controls all downstream targets. Neither is high-specificity signaling—certain DGCs/PDEs show distinct regulatory outputs—organized in a series of small binary modules of interacting DGC/PDE pairs that act independently and in parallel on distinct targets. Rather, most GGDEF/EAL domain proteins of *E. coli* fall into two categories: (i) proteins interconnected by multiple interactions in a c-di-GMP signaling supermodule network (Fig. 1D) and (ii) lonely players that do not engage in contacts with other GGDEF/EAL domain proteins. The latter may contribute to controlling the overall cellular pool of c-di-GMP under some conditions, or they could engage in smaller modules with not-yet-identified c-di-GMP-controlled effector/target systems. In contrast, the supermodule shows a highly interlinked core consisting of DgcE, DgcM, DgcO, PdeR, and PdeG, which, according to criteria for network topography—such as connectivity, the central position on the shortest paths, and betweenness (38)—serve as central nodes or hubs, while several other DGCs and PDEs and a couple of degenerate GGDEF/EAL domain proteins are more peripherally associated (Fig. 1D).

Despite our systematic and unbiased collection of protein interaction data, which did not take into account any previous knowledge about specific protein functions, the hyperconnected five-protein core of the supermodule network turned out to include precisely those components—i.e., DgcE, PdeR, and DgcM—that make up the c-di-GMP-driven switch which turns on CsgD expression and thereby the production of the biofilm matrix components curli and cellulose (Fig. 1D). This is fully consistent with the notion that hubs typically control the flow of information across a network (37, 38), and it also suggests that driving biofilm matrix production is the major and/or most intricately controlled output of c-di-GMP signaling in *E. coli*. The role of the two other multiconnected proteins of the supermodule core, i.e., DgcO and PdeG, is currently less clear, and their input into the c-di-GMP supermodule may be conditional.

Direct interactions between PdeR and DgcM and their key functions for the molecular switch that turns on CsgD expression (Fig. 1E) have been previously analyzed in great detail (13, 14, 39). While it was clear that DgcE acts directly upstream of PdeR and provides the major positive input into this switch (13, 17, 20), the multiple direct protein interactions of DgcE in the c-di-GMP signaling supermodule indicate that this c-di-GMP input is specific and local also. This is further corroborated by the observation that the *dgcE* mutation, just as the *pdeR* and *dgcM* mutations, has a major effect on matrix production (Fig. 3) without eliciting corresponding effects on cellular c-di-GMP levels (Fig. 4A). DgcE could represent an efficient local source of c-di-GMP in the immediate vicinity of the c-di-GMP-sensing trigger PDE PdeR. It is also conceivable that PdeR and DgcE could directly inhibit each other, which would contribute to the positive-feedback loops (Fig. 1E) that confer bistable switch properties to this system (39). Notably, DgcE is one of four DGCs that can interact with PdeR (Fig. 1B), which may result in a dynamic titration equilibrium with changing regulatory outcomes as a function of changing levels of expression and turnover of these proteins (as discussed below). Both DgcE’s central function in turning on biofilm matrix production and its multidomain structure, which allows multiple interactions and most likely also allows perception of multiple signals, clearly warrant future specific analyses of this most complex of all GGDEF/EAL domain proteins in *E. coli*.

### Homomeric and heteromeric interactions between GGDEF domains that may provide novel unorthodox regulatory mechanisms.

It is now widely accepted that the GGDEF domains of DGCs have to dimerize to allow the condensation of two GTP to form c-di-GMP and that, in general, DGC activity is controlled by N-terminal signal input domains that can promote this homodimerization (40). Even DgcZ, whose “default” state is as an active dimer that can be inhibited by zinc binding, dimerizes via its N-terminal zinc-binding domain (41). Accordingly, the isolated GGDEF domains of 10 of the 12 DGCs of *E. coli* are unable to dimerize on their own (Fig. 1C). However, there are two interesting exceptions to this rule: DgcM and DgcO. The purified GGDEF^DgcM^ domain alone indeed shows DGC activity *in vitro* (13). The potential for unassisted homodimerization of the GGDEF^DgcM^ and GGDEF^DgcO^ domains also suggests that the associated N-terminal domains play a role that is different from that of the sensory input domains of other DGCs. For instance, these N-terminal domains in DgcM and DgcO may be inhibitory rather than activating or, alternatively, they may contact other proteins. Thus, the two PAS domains of DgcM (13) and the oxygen-sensing globin sensor domain of DgcO (16) are directly involved in the interactions with the GGDEF^PdeR^ and EAL^PdeR^ domains (Fig. 1B and Fig. S3). Furthermore, two of the seven degenerate GGDEF domains in *E. coli*, i.e., GGDEF^PdeR^ and GGDEF^PdeK^, showed a strong potential to homodimerize as isolated domains. Very weak cryptic DGC activity for PdeR, which should depend on this GGDEF^PdeR^ homodimerization, has been observed before (13). The formation of homodimers of GGDEF^PdeR^ and of GGDEF^PdeK^ may also control the PDE activity of their EAL domains.

In addition, strong heteromeric interactions between GGDEF domains (>90% strength relative to the reference RpoS/RssB interaction) were observed for a highly nonrandom pattern of 12 pairs among the 171 possible heteromeric combinations (also found in vector swaps, except for GGDEF^CdgI^ interactions, since overproduction of GGDEF^CdgI^ from the higher-copy-number vector was toxic; Fig. 1C). In cases in which these heteromeric interactions involve degenerate GGDEF domains of PDEs and the intact GGDEF domains of DGCs, similar interactions can in general also be seen in the presence of additional domains (see, e.g., interactions of PdeK with several DGCs in Fig. 1A). In cases in which this correspondence is not found, intramolecular domain interactions may occlude possible intermolecular contacts of GGDEF domains. Such a previously described case is the functionally important interaction of GGDEF^DgcM^ with MlrA, which in a 2H assay can be seen only in the absence of the N-terminal PAS domain of DgcM (13).

What could be the functional role of this potential for efficient heterodimerization between a few distinct GGDEF domains? Heteromer formation could be a regulatory mechanism that operates by competing with homodimerization of GGDEF domains. If at least one of the partners is a canonical GGDEF domain (with intact A-site), this may directly affect its DGC activity. For degenerate GGDEF domains such as GGDEF^PdeK^ that are linked to a canonical EAL domain, not only homodimerization but also heterodimerization may have an impact on the PDE activity of the associated canonical EAL domains. A noteworthy case seems to be that of CdgI, a degenerate GGDEF domain protein of hitherto-unknown function with a clear potential for strong interactions with several other GGDEF domains (Fig. 1C). Under standard laboratory conditions, CdgI is not expressed (24) and overexpression of GGDEF^CdgI^ is toxic (Fig. 1A and C). Under the still-unknown conditions that drive its physiological expression, CdgI may inhibit some DGCs or degenerate GGDEF domain proteins by engaging their GGDEF domains in heteromeric interactions. Moreover, CdgI has an intact I-site and binds c-di-GMP (Fig. S4), which raises the possibility that these CdgI interactions could be modulated by c-di-GMP itself, which could establish interesting negative or positive regulatory feedback loops.

Finally, a specific heteromeric contact was observed for GGDEF^DgcT^ and the degenerate GGDEF^CsrD^ domain (Fig. 1C). This is noteworthy since CsrD also plays an indirect negative regulatory role in the expression of the *dgcT* gene (via the small RNAs CsrB and CsrD and the mRNA binding protein CsrA) (28, 42). Thus, CsrD may exert dual control over DgcT by indirectly inhibiting its expression and by directly interacting with the protein.

### Expression and coregulation patterns of DGCs, PDEs, and degenerate GGDEF/EAL domain proteins.

For protein interactions to occur, partner proteins have to be coexpressed under physiological conditions. Cellular levels of GGDEF/EAL domain proteins of *E. coli* differ by more than 3 orders of magnitude (Fig. 2) and, with respect to regulation, they fall into two large classes: (i) proteins already expressed during vegetative growth and (ii) proteins under the control of the stationary-phase sigma factor RpoS. Whereas cellular levels of the former decrease during entry of the stationary phase, levels of the latter increase (Fig. 2). Thus, cellular ratios between GGDEF/EAL domain proteins change drastically along the growth cycle of *E. coli*. Notably, DgcE, DgcM, and PdeR, as well as DgcO and PdeG, i.e., the DGCs and PDEs that make up the multiconnected core of the c-di-GMP control supermodule, are all under RpoS control (17, 20, 24). They are thus coexpressed with each other as well as with the transcription factors MlrA and CsgD (Fig. 2A), which mediate the output, i.e., biofilm matrix production.

Furthermore, direct interactions of proteins and their regulatory consequences are highly sensitive to stoichiometry. Thus, only proteins in excess can efficiently inhibit by direct interaction, therefore making more likely the hypotheses in which a highly expressed protein such as DgcO efficiently inhibits the function of its potential partner proteins rather than vice versa. Finally, massive degradation was observed for DgcE and PdeA, with the size of the detectable proteolytic fragments suggesting cleavage between the domains mediated by a currently unknown protease. This proteolysis could actually lead to a massive underestimation of the effective cellular levels of these proteins, if cleavage also occurs between the GGDEF domain and the C-terminal EAL domain which carries the tag for detection. This is consistent with both DgcE and PdeA belonging to those proteins with a high transcription/protein ratio (Fig. 2B). Overall, cellular levels of DgcE and PdeA seem highly dynamic due to control of expression as well as of turnover. For PdeA, this instability may contribute to its rapid replacement by the structurally highly related PdeF during the transition to the anaerobic conditions which induce PdeF (29).

### The "fountain" model: a novel concept of local c-di-GMP signaling and the key role of the master PDE PdeH.

When *E. coli* is grown in complex LB medium, c-di-GMP is undetectable during rapid growth. During the postexponential and early stationary phases (43), c-di-GMP reached detectable but still relatively low levels (approximately 1.4 pmol/mg cellular protein, which corresponds to approximately 80 nM; Fig. 4A). In contrast, a mutant lacking PdeH—with up to several thousand molecules, it is the major PDE *in E. coli* (Fig. 2A)—had a strongly elevated cellular c-di-GMP level (approximately 11 pmol/mg cellular protein or 0.66 μM; Fig. 4A); it was mainly DgcE that contributed to the elevation in level, but several other DGCs (except DgcC and DgcT) also did so to minor degrees (Fig. 4B). This observation has important implications: (i) *E. coli* cells actively maintain an unexpectedly low c-di-GMP level even under conditions in which c-di-GMP-dependent processes are activated; (ii) they do so in a highly dynamic equilibrium in which PdeH counteracts the DGCs that are expressed; and (iii) these DGCs are in an active state—i.e., activation via their N-terminal domains does not need any rare or exotic signals.

PdeH is thus a globally acting PDE that antagonizes the global effects of several DGCs, i.e., their potential to significantly contribute to the highly dynamic cellular c-di-GMP pool. This is most pronounced for DgcE—a *dgcE* mutation does not alter the cellular c-di-GMP level in the presence of PdeH but reduces cellular c-di-GMP levels 4-fold in a *pdeH* mutant background (Fig. 4A and B). By acting as a powerful drain with respect to the overall cellular c-di-GMP pool, PdeH could thus restrict the ability of several DGCs to serve as specific local c-di-GMP sources in the immediate vicinity of particular c-di-GMP-binding effectors. Thereby, these colocalized DGCs would overcome the conundrum that the dissociation constant (*K*_*d*_) value for c-di-GMP binding by various effectors is usually in the high nanomolar to low micromolar range (44, 45), i.e., at least severalfold higher than the global cellular c-di-GMP level. In other words, the globally acting PdeH sets the conditions that allow various DGCs to engage in specific local signaling—like small fountains at the bottom of a pool whose presence and specific action are revealed only at a low water level in the pool (Fig. 4C).

This new concept can also explain why no specific antagonistic DGC/PDE pairs teaming up in complexes were observed (Fig. 2)—instead, the strongly expressed PdeH globally antagonizes the action of all DGCs. Consistent with this key role and unlike many other GGDEF/EAL domain proteins, PdeH was found to be extremely conserved in 61 genomes of *E. coli* that represent the major phylogenetic groups and pathotypes (6). It is noteworthy that PdeH consists of a highly active stand-alone EAL domain without an N-terminal sensor domain, indicating that its activity is just a function of its high cellular level. In contrast, the roles of other PDEs seem more specific and seem to be regulated by sensory input via their N-terminal domains. As peripheral interaction partners, some may conditionally affect the output of the core “supermodule” network (Fig. 1D). Some may act as controllable local sinks of c-di-GMP in the immediate vicinity of an effector/target system in a manner reminiscent of local cyclic AMP (cAMP) signaling driven by specific cAMP phosphodiesterases in eukaryotes (46, 47). Finally, some may function like PdeR (13) (Fig. 1E) and PdeL (48), which are trigger PDEs that use their ability to bind and cleave c-di-GMP as regulatory input to control their functional interactions with specific target proteins or DNA (14).

Notably, this novel concept of local c-di-GMP signalling based on active maintenance of a low cellular c-di-GMP level does not require compartmentalization with separate “local pools” of c-di-GMP. Instead, it relies on the increased probability of binding of c-di-GMP molecules to an effector if this c-di-GMP is produced by a local source in the immediate vicinity of the effector. Indeed, several DGCs of *E. coli* have been found in protein complexes with effector/target components as follows: (i) DgcM (13, 14) and DgcE (see above) engage in a complex with PdeR and MlrA to control *csgD* transcription; (ii) DgcO is a member of a specific degradosome complex that also includes the c-di-GMP-controlled PNPase (15, 16); and (iii) DgcN, when activated by transmembrane signaling, localizes to the Z-ring, where it interacts with FtsZ and ZipA to interfere with cell division (49). Identifying all c-di-GMP-binding effector/targets directly contacted by locally acting DGCs will be a challenge for future studies. An obvious candidate is cellulose synthase, which is activated by c-di-GMP binding via the PilZ domain of the BcsA subunit (50, 51). Without affecting the cellular c-di-GMP level, the *ΔdgcC* mutation specifically eliminates cellulose biosynthesis but does not affect curli fiber production (Fig. 3). Even in a *pdeH* mutant background, with its elevated c-di-GMP levels, DgcC does not contribute to the cellular c-di-GMP pool (Fig. 4B) and yet remains essential for cellulose biosynthesis (Fig. S7).

In conclusion, our systematic analysis of all 29 GGDEF/EAL domain proteins has provided novel evidence for highly specific local c-di-GMP signaling by (i) revealing a tightly interconnected protein network of a specific subset or “supermodule” of DGCs and PDEs that also show functional and regulatory association and (ii) demonstrating a lack of correlation between global cellular c-di-GMP levels and the phenotypes generated by knocking out these DGCs and PDEs. Our results led to a novel concept of local c-di-GMP signaling and, as discussed above, also provide the basis for numerous detailed hypotheses on the molecular mechanisms involved. These can now guide future studies that will focus on particularly interesting members of this network, such as, e.g., DgcE, DgcO, and CdgI and also DgcC, with its highly specific control of cellulose biosynthesis.

## MATERIALS AND METHODS

### Bacterial strains and growth conditions.

The strains used were derivatives of *E. coli* K-12 strains W3110 (52) and AR3110 (33), with the latter being a direct derivative of W3110 in which codon 6 in the chromosomal copy of *bcsQ* (which in the cellulose-negative W3110 is the stop codon TAG) was changed to the sense codon TTG (33). Where required, this intact copy of *bcsQ* was transferred using a nearby insertion of a kanamycin resistance cassette (*kan*; inserted between *dppF* and *yhjV*), which is >90% P1 cotransducible with *bcsQ*^*wt*^ (A. Richter and R. Hengge, unpublished data) and which can be flipped out using FLP recombinase (53).

The mutations in all GGDEF/EAL domain-encoding genes (except *pdeL*) are full *orf* deletion/resistance cassette insertions generated in W3110 and were previously described (17, 20, 24). *pdeL*::*cat* is a full *orf* deletion/resistance cassette insertion constructed via one-step inactivation (53) using oligonucleotide primers listed in Table S1 in the supplemental material. The single-copy *csgB*::*lacZ* reporter fusion was described before (20) and was introduced into the *att*(λ) location of the chromosome of a W3110 derivative containing a Δ*lac*(*I-A*)::*scar* deletion via phage λRS45 (54). Single lysogeny was tested by a PCR approach (55).

10.1128/mBio.01639-17.8 TABLE S1  Oligonucleotide primers used in the present study. Download TABLE S1, PDF file, 0.1 MB. Copyright © 2017 Sarenko et al. 2017 Sarenko et al. This content is distributed under the terms of the Creative Commons Attribution 4.0 International license.

C-terminally 3× FLAG-tagged chromosomally encoded constructs were generated using plasmid pSUB11 as a PCR template and the oligonucleotide primers listed in Table S1 following a procedure based on λRED technology (56). The primer combinations used for C-terminal 3× FLAG tagging of the genes encoding PdeR and the MlrA transcription factor were *yciR-*FLAG-f/*yciR*-FLAG-r and *mlrA-*FLAG-f/*mlrA*-FLAG-r, respectively (13). Chromosomal C-terminal 3× FLAG tagging of *csgD* was done in W3110 using primers that were previously published (57).

FLP recombination target (FRT)-flanked resistance cassettes initially introduced in various genetic constructs were eliminated using FLP recombinase (53), and the relevant chromosomal regions of the resulting mutants were verified by DNA sequencing of PCR fragments (GATC Biotech).

Cells were grown in LB medium (58) under conditions of aeration at 28°C with antibiotics added as recommended to ensure maintenance of plasmids. Macrocolonies were grown at 28°C for 5 days on salt-free LB agar plates supplemented with Congo red (CR) and Coomassie brilliant blue as previously described (34).

### Stereomicroscopy.

*E. coli* macrocolony biofilms were visualized at ×10 magnification with a Stemi 2000-C stereomicroscope (Zeiss; Oberkochen, Germany). Digital photographs were taken with an AxioCam ICC3 digital camera coupled to the stereomicroscope, which was operated using AxioVision 4.8 software (Zeiss).

### Detection of protein-protein interactions *in vivo* using a bacterial two-hybrid system.

The Bacterio-Match II two-hybrid system (Agilent Technologies) uses hybrid proteins linked to the NTD of lambda cI (expressed from pBT) and to the bacterial RNA polymerase alpha-NTD (expressed from pTRG), with coexpression of interacting proteins leading to expression of the *HIS3* gene. This suppresses histidin auxotrophy of the *E. coli* reporter strain (a derivative of XL1-Blue MRF′) in a manner that can be fine-tuned by adding the His3 inhibitor 3-amino-1,2,4-triazole (3-AT) (21). If not indicated otherwise, hybrid proteins included the entire sequence of cytoplasmic GGDEF/EAL domain proteins or the entire cytoplasmic parts of those proteins with an N-terminal membrane-inserted domain. Hybrid proteins containing specific domains only also included the natural linker regions upstream and downstream of the respective domains to allow proper folding and flexibility.

The assay procedure was performed according to the instruction manual of the Bacterio-Match II two-hybrid system vector kit with slight alterations. In particular, cotransformation of plasmids was performed by heat shock with approximately 24 fmol of the plasmids and 50 µl of competent cells in half the volumes as indicated in the manual. To counteract basal expression (occurring in the absence of interacting hybrid proteins) potentially leading to growth on selective minimal media (lacking histidine), a 5 mM concentration of the competitive inhibitor 3-AT was added to the plates. For a qualitative readout, cotransformants first obtained on nonselective plates can be patched on selective plates, with growth of the patches being only visually assessed. Alternatively, for a more quantitative readout, cotransformant colonies can be obtained and counted directly on selective plates relative to nonselective plates (21). For the latter method, cotransformants (200 µl after 1:20 or 1:40 dilution) were plated on nonselective screening plates containing M9 salts, 0.4% glucose, 50 µM IPTG (isopropyl-β-d-thiogalactopyranoside), 25 µg/ml chloramphenicol, 12.5 µg/ml tetracycline, and several supplements as described in the manual. In parallel, 200 µl was plated on selective plates also containing 3-AT. When cells grew insufficiently even on nonselective plates, they were plated at a higher titer. Cells on nonselective plates were grown for 24 h at 37°C. Cells on selective plates were grown for 24 h at 37°C followed by 48 h at 28°C. The colonies obtained were counted, and the ratio of the CFU counts on selective plates to the counts on nonselective plates was calculated. Interaction activity was normalized to the strong and well-studied interaction of the sigma factor RpoS and its proteolytic targeting factor RssB (23), which was assayed in parallel in each series of experiments. Each combination of any two proteins in the 2H experiments was tested in at least two independent biological replicates. Interactions were considered significant that showed a reproducible strength of >20% of that of the RpoS/RssB interaction if seen in only one of the two vector combinations (due to steric hindrance, interaction is not always reciprocal in a vector swap) or that showed reproducible strengths of >10% and >2% of that of the RpoS/RssB reference pairs if the interaction was detected with both of the reciprocal vector combinations.

### Protein purification.

C-terminally 3× FLAG-tagged PdeL, for use as the standard for protein quantification on immunoblots, was purified from a pCAB18-derived plasmid (31) in a W3110 derivative carrying *lacI*^q^Δ*lacZYA*::*scar* and Δ*pdeL*:*:scar* mutations. Cells were grown at 37°C to an OD_578_ of 0.1, IPTG (0.1 mM) was added, and incubation proceeded for 3.5 h. Cells were harvested and resuspended in immunoprecipitation (IP) buffer (100 mM Tris HCl [pH 8], 300 mM NaCl, 1% Triton X-100) that included proteinase inhibitor phenylmethylsulfonyl fluoride (PMSF) (1 mM) and were sonicated in a cooling bath. Insoluble material was removed by centrifugation. IP buffer/PMSF was added to the supernatant to reach a final volume of 750 µl, and the reaction mixture was mixed with 250 µl of a magnetic microparticle suspension (Sigma) for preadsorption and incubated for 3 h at 4°C under conditions of rotation. The tubes were placed in a magnetic separator, and the supernatant was transferred into a new tube containing 50 µl (packed gel volume) of an anti-FLAG M2 magnetic bead suspension (Sigma) previously equilibrated once in TBS (50 mM Tris HCl [pH 7.5], 150 mM NaCl) and twice in TBS–5% milk powder (TBSM). The immunoprecipitation of FLAG fusion protein was carried out overnight at 4°C under conditions of rotation. Washing steps and elution of FLAG fusion protein from the magnetic beads by competition with the 3× FLAG peptide (Sigma) were performed according to the anti-FLAG M2 magnetic bead technical bulletin (Sigma).

The N-terminally His6-tagged cytoplasmic GGDEF domain of CdgI was purified from a plasmid derived from pQE30xa (Qiagen) in *E. coli* Fi8202 (59). Strains were grown in LB-ampicillin at 37°C to an OD_578_ of 0.5 to 0.7, IPTG (1 mM) was added, and incubation continued for 4 h. Cells were harvested and lysed by passage through a French press. The soluble protein fraction was used for protein purification according to a standard protocol (QIAexpressionist manual; Qiagen). Proteins were dialyzed in diguanylate cyclase reaction buffer (25 mM Tris-HCl [pH 8], 100 mM NaCl, 10 mM MgCl_2_, 5 mM β-mercaptoethanol, 5% glycerol).

### SDS polyacrylamide gel electrophoresis, immunoblot detection, and quantification of proteins.

For immunoblot analyses of 3× FLAG-tagged proteins, samples were taken at different time points during growth from cultures grown in LB medium (at OD_578_ levels of 0.3, 1, and 2.5 and after 11 or 12 h of incubation and overnight [oN] incubation). Samples corresponding to up to 320 µg of total cellular protein were precipitated with 10% trichloroacetic acid (TCA) for at least 20 min on ice. After washing was performed once with ice-cold acetone, the protein pellets were resuspended in SDS-PAGE sample buffer and incubated for 10 min at 70°C and for 10 min at 100°C. Samples were adjusted such that all samples derived from the same culture had similar amounts of total protein. These were then loaded along with different concentrations of the purified standard protein (3× FLAG-tagged PdeL, used for protein quantification) and run on 10% SDS–polyacrylamide gels. 3× FLAG fusion proteins were detected by immunoblotting as previously described (60) using an antibody against FLAG tag (Sigma) and anti-mouse IgG horseradish peroxidase (HRP) conjugate from donkey (GE Healthcare). Use of the WesternC Precision Plus marker (Bio-Rad) also required the addition of StrepTactin-HRP conjugate. Proteins were visualized using Western Lightning Plus-ECL enhanced chemiluminescence substrate (PerkinElmer). Nonspecific protein bands were identified by comparison of the band patterns of recombinant strain samples to those of wild-type *Escherichia coli* K-12 samples with similar amounts of cellular protein loaded on the same gels. 3× FLAG-tagged proteins in cellular extracts were quantified using ImageJ image analysis software (61).

### Detection of c-di-GMP binding to proteins by UV cross-linking.

Binding of [α-^32^P]c-di-GMP (42 nM, 6,000 Ci/mmol) or [α-^32^P]GTP (42 nM, 3,000 Ci/mmol) to purified proteins *in vitro* was assayed by UV cross-linking according to the method described in reference 62. Radiolabeled nucleotides were obtained from Hartmann Analytic GmbH. Proteins were separated in 15% SDS polyacrylamide gels.

### Determination of cellular c-di-GMP levels.

Strains were grown at 28°C under conditions of aeration in LB medium. At the indicated OD_578_ level, culture samples were harvested, concentrated into 1.5 ml LB, and pelleted again (4°C, 12,700 rpm, 10 min). Sample extraction and analysis of c-di-GMP by liquid chromatography/tandem mass spectrometry (LC-MS/MS) were performed as described previously (43). Intracellular levels of c-di-GMP were normalized to the corresponding total amount of cellular protein determined using a Pierce bicinchoninic acid (BCA) protein assay kit (Thermo Scientific). Extractions were performed in biological triplicates.

### Determination of β-galactosidase activity.

β-Galactosidase activity was assayed by the use of *o*-nitrophenyl-b-d-galactopyranoside (ONPG) as a substrate and is reported as millimoles of *o*-nitrophenol per minute per milligram of cellular protein (58). Data shown represent average values with standard deviations indicated as obtained from three biological replicates.

### Software tools.

Protein sequence alignments were performed with Clustal Omega and phylogenetic analyses with Simple phylogeny (both accessible at http://www.ebi.ac.uk/tools).
