# Supplementary figures and images for: More than Enzymes That Make or Break Cyclic Di-GMP—Local Signaling in the Interactome of GGDEF/EAL Domain Proteins of Escherichia coli
Source: mBio. 2017 Oct 10;8(5):e01639-17. doi: 10.1128/mBio.01639-17 (PMC5635695; doi:10.1128/mBio.01639-17)

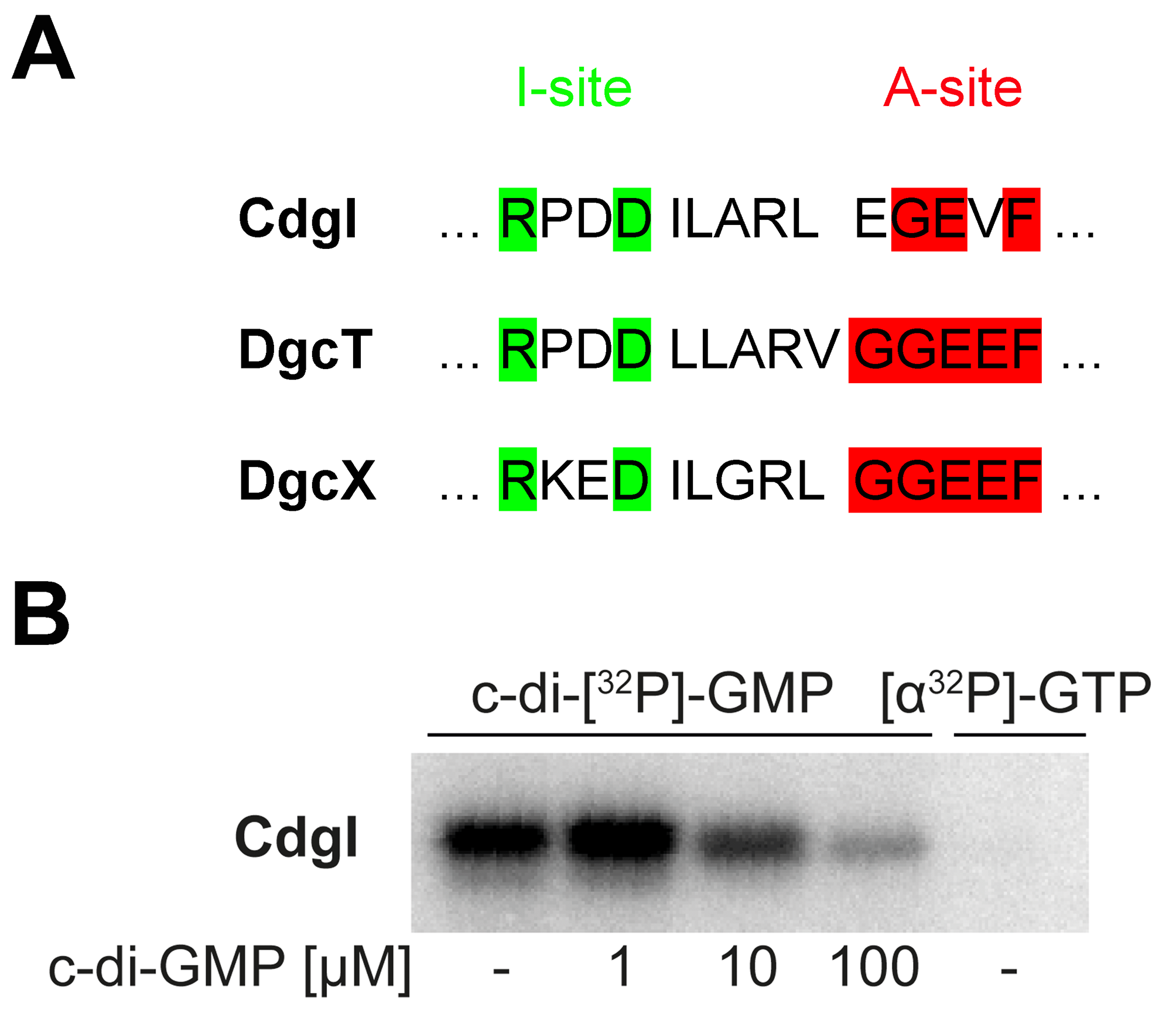

Supplement: FIG S4 [file mbo005173526sf4.tif]

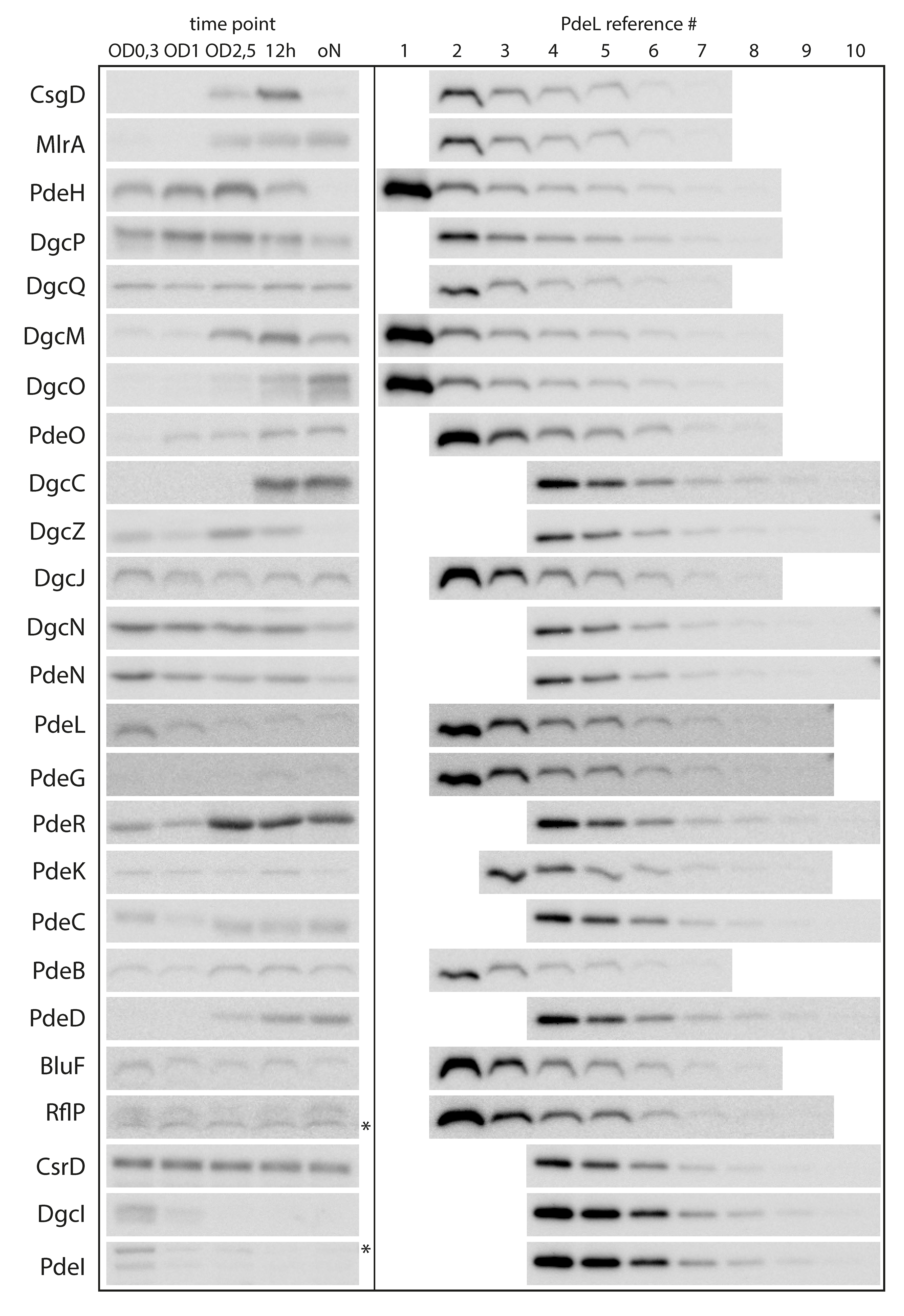

Supplement: FIG S5 [file mbo005173526sf5.tif]

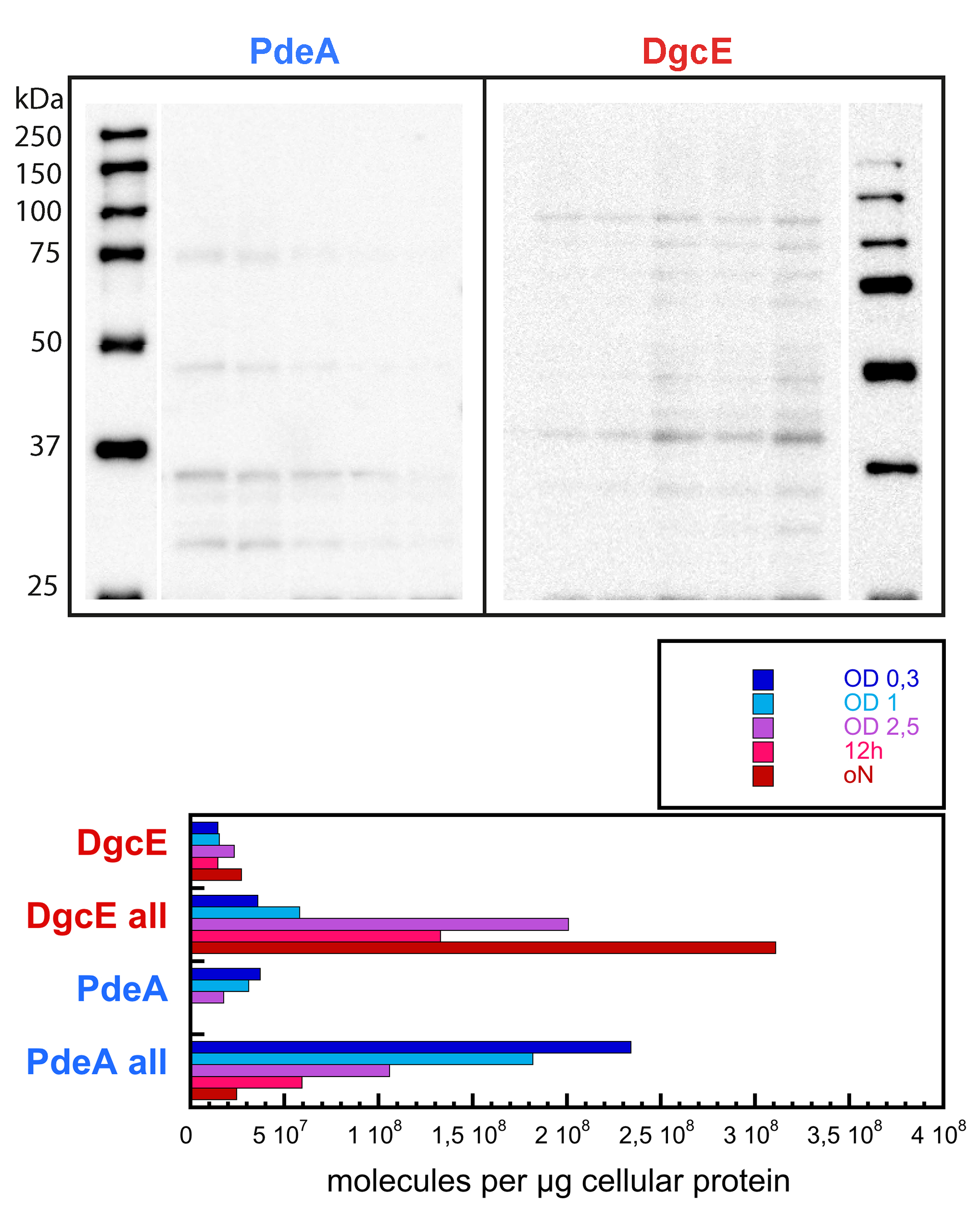

Supplement: FIG S6 [file mbo005173526sf6.tif]

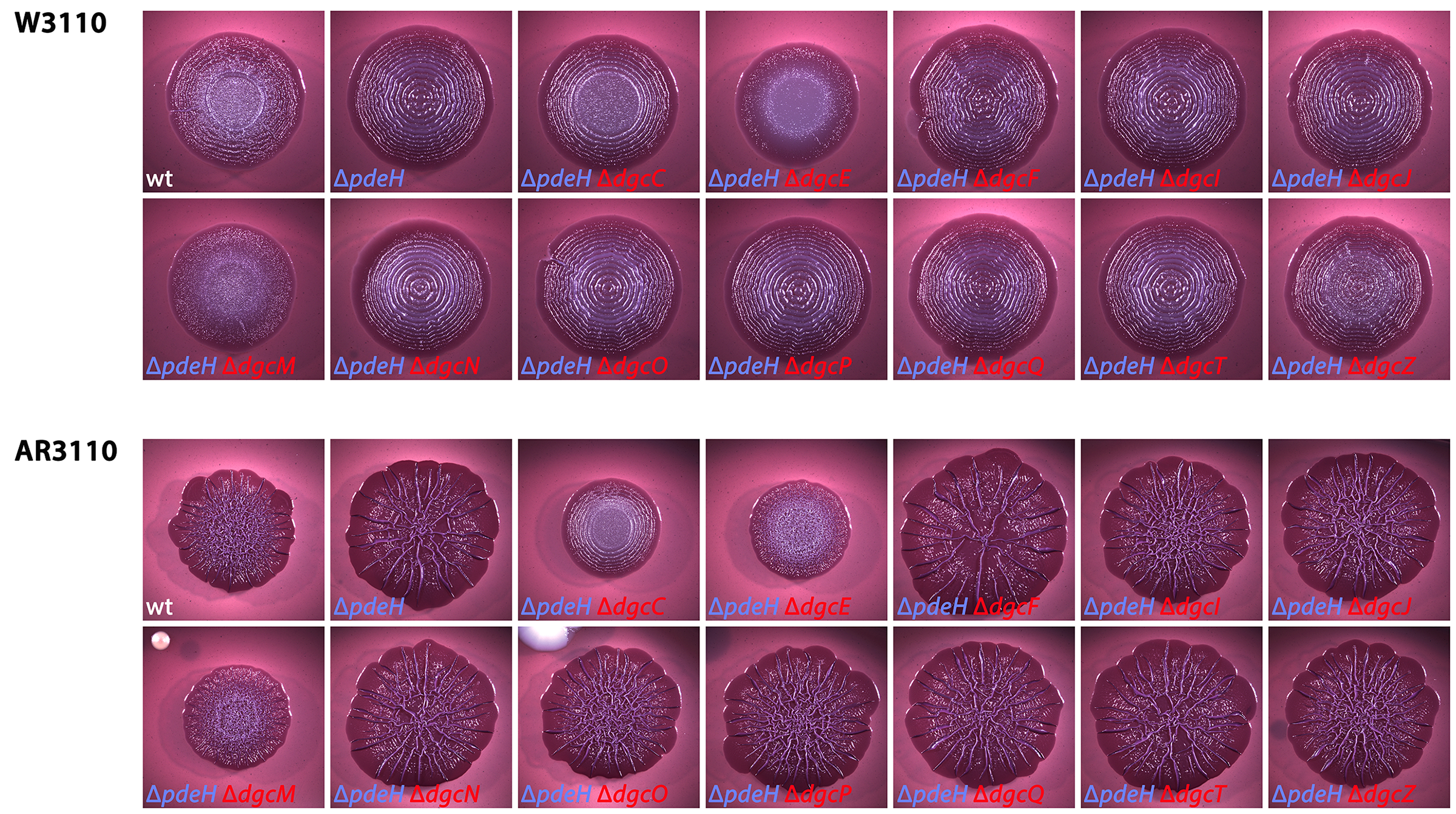

Supplement: FIG S7 [file mbo005173526sf7.tif]
