# Supplementary material for: More than Enzymes That Make or Break Cyclic Di-GMP—Local Signaling in the Interactome of GGDEF/EAL Domain Proteins of Escherichia coli
Source: mBio. 2017 Oct 10;8(5):e01639-17. doi: 10.1128/mBio.01639-17 (PMC5635695; doi:10.1128/mBio.01639-17)
Supplement: TABLE S1 [file mbo005173526st1.pdf]

Supporting S1 Table

Oligonucleotide primers used in the present study[1].

I. Primers used for cloning cytoplasmic parts of all GGDEF, EAL and degenerate GGDEF/EAL domain proteins as well as GGDEF domains alone into pBT and pTRG:

dgcC-pBT-EcoRI-for	5`-GCC**GAATTC**CCTGGCGGAACATAAACGCAGG-3`
dgcC-BglII-rev	5`-GCC**AGATCT**TCAGGCCGCCACTTCGGTGC-3`
dgcC-pTRG-EcoRI-for	5`-GCC**GAATTC**TCCTGGCGGAACATAAACGCAGG-3`
dgcE-pTRG-EcoRI-for	5`-GCC**GAATTC**TTGAGCTAAAACGCACCGAACAGG-3`
dgcE-XhoI-rev	5`-GCC**CTCGAG**TCAGTTAATCGCGAAATAACTAC-3`
dgcF-pBT-EcoRI-for	5`-GCC**GAATTC**TCTAATGAAGCAAGTTGCGCTG-3`
dgcF-XhoI-rev	5`-GCC**CTCGAG**TCAGACAACTTCCTCACCG-3`
dgcF-pTRG-EcoRI-for	5`-GCC**GAATTC**TTCTAATGAAGCAAGTTGCGCTG-3`
dgcI-pBT-EcoRI-for	5`-GCC**GAATTC**TGTGATTAATCGTGGTATTGTG-3`
dgcI-XhoI-rev	5`-GCC**CTCGAG**TTACTGCCAGCTAATCTGTGC-3`
dgcI-pTRG-EcoRI-for	5`-GCC**GAATTC**TTGTGATTAATCGTGGTATTGTG-3`
dgcJ-NotI-for	5`-GAA**GCGGCCGC**TCGGATGCACTTCCGTTTATACC-3`
dgcJ-XhoI-rev	5`-GCC**CTCGAG**TCATGAACGGCTGTTTTTGTTCTGC-3`
dgcN-NotI-for	5`-GAA**GCGGCCGC**TCACAATGGCCTGGTAGAAGCG-3`
dgcN-XhoI-rev	5`-GCC**CTCGAG**TTATCTCACCAGCTTTTCGGC-3`
dgcO-NotI-for	5`-GAA**GCGGCCGC**TGAGATGTATTTTAAAAGAATG-3`
dgcO-XhoI-rev	5`-GCC**CTCGAG**CTAAAGACTGGCTTTCCAGAG-3`
dgcP-pBT-EcoRI-for	5`-GCC**GAATTC**TTCAGATCAGATTATCGCC-3`
dgcP-XhoI-rev	5`-GCC**CTCGAG**TCAGGAATGTAGCGCTGG-3`
dgcP-pTRG-EcoRI-for	5`-GCC**GAATTC**TTTCAGATCAGATTATCGCC-3`
dgcQ-pBT-EcoRI-for	5`-GCC**GAATTC**TGTTAGCAACATGTATGTTCTG-3`
dgcQ-XhoI-rev	5`-GCC**CTCGAG**TTAAGCGTTATCGCTCGCGAATACC-3`
dgcQ-pTRG-EcoRI-for	5`-GCC**GAATTC**TTGTTAGCAACATGTATGTTCTG-3`
dgcT-pBT-EcoRI-for	5`-GCC**GAATTC**TAAGAACATTGCACATCGCGATCCC-3`
dgcT-XhoI-rev	5`-GCC**CTCGAG**TTATGGTGACTCACAAAAATTCACC-3`
dgcT-pTRG-EcoRI-for	5`-GCC**GAATTC**TTAAGAACATTGCACATCGCGATCCC-3`
dgcZ-pBT-EcoRI-for	5`-GCC**GAATTC**TATCAAGAAGACAACGGAAATTG-3`
dgcZ-XhoI-rev	5`-GCC**CTCGAG**TTAAACTCGGTTAATCACATTTTG-3`
dgcZ-pTRG-EcoRI-for	5`-GCC**GAATTC**TTATCAAGAAGACAACGGAAATTG-3`
pdeA-pBT-EcoRI-for	5`-GCC**GAATTC**ACATCGCAGTGAATGGCTTAATCGC-3`
pdeA-XhoI-rev	5`-GCC**CTCGAG**TTAATCAGCTAATGGCTGCGGGCG-3`
pdeA-pTRG-EcoRI-for	5`-GCC**GAATTC**CACATCGCAGTGAATGGCTTAATCGC-3`
pdeB-pBT-EcoRI-for	5`-GCC**GAATTC**ACGCCGTATTCAGTCACCGCATC-3`
pdeB-XhoI-rev	5`-GCC**CTCGAG**TCACAAATGTTGCTCGGCCCAGCG-3`
pdeB-pTRG-EcoRI-for	5`-GCC**GAATTC**CACGCCGTATTCAGTCACCGCATC-3`
pdeC-pBT-EcoRI-for	5`-GCC**GAATTC**ACTACGTATTCGACAAAACTATTTATCTCCC-3`
pdeC-XhoI-rev	5`-GCC**CTCGAG**TTATTGCCCGCGCGTTAACTCCCG-3`

[1] Relevant nucleotides (e.g. restriction sites, mutations introduced or pKD45-specific sequences) labeled in **bold**. All primer sequences are given from 5´- to 3´-end.

pdeC-pTRG-EcoRI-for	5`-GCC**GAATTC**CACTACGTATTCGACAAAACTATTTATCTCCC-3`
pdeD-pBT-EcoRI-for	5`-GCC**GAATTC**CGCCTGGCTGGCGACCGCTTACCG-3`
pdeD-XhoI-rev	5`-GCC**CTCGAG**TTAACGTAACGGCATAATGGG-3`
pdeD-pTRG-EcoRI-for	5`-GCC**GAATTC**AGGCCTGGCTGGCGACCGCTTACCG-3`
pdeF-pBT-EcoRI-for	5`-GCC**GAATTC**ACGTCAGAGAGCGGTAGTCAGACGC-3`
pdeF-pBT-XhoI-rev	5`-GCC**CTCGAG**TCAGGCACTTTCGCGAATGGG-3`
pdeF-pTRG-EcoRI-for	5`-GCC**GAATTC**AACGTCAGAGAGCGGTAGTCAGACGC-3`
pdeG-NotI-for	5`-GAA**GCGGCCGC**AGATAGATATTTCAATAAAAGC-3`
pdeG-XhoI-rev	5`-GCC**CTCGAG**TCACTCAACCACAACCTTCACC-3`
pdeI-NotI-for	5`-GAA**GCGGCCGC**TCGCTGGCGTTTTTGCCTGACTCAACGG-3`
pdeI-XhoI-rev	5`-GCC**CTCGAG**CTACTCTTTTACTAATTTTCC-3`
pdeK-pBT-EcoRI-for	5`-GCC**GAATTC**CGCCATCCCAGCCAAGGAGCTTGTTGG -3`
pdeK-XhoI-rev	5`-GCC**CTCGAG**CTACTTTTCTTCCAGGTA-3`
pdeK-pTRG-EcoRI-for	5`GCC**GAATTC**AGGCCATCCCAGCCAAGGAGCTTGTTGG-3`
pdeL-NotI-for	5`-GAA**GCGGCCGC**AAATTCATGTGATTTTCGTG-3`
pdeL-SpeI-rev	5`-CCG**ACTAGT**CTATCAACCACCTGCTTTCATTA-3`
pdeN-pBT-EcoRI-for	5`-GCC**GAATTC**TAGCGTACGTATGCGCCCCGGC-3`
pdeN-XhoI-rev	5`-GCC**CTCGAG**TTACCACTGCGGCGTATACGG-3`
pdeN-pTRG-EcoRI-for	5`- GCC**GAATTC**CAAGCGTACGTATGCGCCCCGGC -3`
pdeO-NotI-for	5`-GAA**GCGGCCGC**AAAGCTAACCGATGCGGATAATGCC-3`
pdeO-XhoI-rev	5`-GCC**CTCGAG**TCAGATTTTCAGCGGTAACACGC-3`
cdgI-pBT-EcoRI-for	5`-GCC**GAATTC**ACAGGAGCTGCAACTCTCCAGC-3`
cdgI-XhoI-rev	5`-GCC**CTCGAG**TTAAATATGATGAATAATCAC-3`
cdgI-pTRG-EcoRI-for	5`-GCC**GAATTC**AACAGGAGCTGCAACTCTCCAGC-3`
csrD-pBT-EcoRI-for	5`-GCC**GAATTC**ACTGGCGGTACGCTGGTTACAACGC-3`
csrD-pBT-XhoI-rev	5`-GCC**CTCGAG**TTAAACCGAGTATCTTTGTG-3`
csrD-pTRG-EcoRI-for	5`-GCC**GAATTC**AACTGGCGGTACGCTGGTTACAACGC-3`
rflP-pBT-EcoRI-for	5`-GCC**GAATTC**AAAGATTTTTTTGGAGAAT-3`
rflP-XhoI-rev	5`-GCC**CTCGAG**TTATCGCTGAACCAACGTCG-3`
rflP-pTRG-EcoRI-for	5`-GCC**GAATTC**AAAAGATTTTTTTGGAGAAT-3`
dgcE-3xPAS-XhoI-rev	5`-GCTA**CTCGAG**TTACAGCAGGATACGTTCCTG-3`
dgcE-GGDEF-pTRG-EcoRI-for	5`-GTAC**GAATTC**CCTCACGCAAAATGCTGCGCCAG-3`
dgcE-GGDEF-XhoI-rev	5`-GCTA**CTCGAG**ATCAAGCGACATCGCCGC-3`
dgcE-EAL-pBT-EcoRI-for	5`-GCC**GAATTC**AGTTTACGAACCGCAGCAAGC-3`
dgcE-EAL-pTRG-EcoRI-for	5`-GCC**GAATTC**AAGTTTACGAACCGCAGCAAGC-3`
dgcN-HAMP-XhoI-rev	5`-GCC**CTCGAG**TTAGTATTTGAAATTATCGCCATC-3`
dgcN-GGDEF-NotI-for	5`-GAA**GCGGCCGC**TGAGTGGCAGCTTCGTTTACAG-3`
dgcO-GS-XhoI-rev	5`-GCC**CTCGAG**CTAGTTATGGCCCCACGTATCGTT-3`
dgcO-GGDEF-NotI-for	5`-GAA**GCGGCCGC**TAACAGAAGTCTGCGGGTGAAA-3`
dgcP-GGDEF-pBT-EcoRI-for	5`- GCC**GAATTC**CATTGCGCAATCGTATACCGAC -3`
dgcP-GGDEF-	5`- GCC**GAATTC**CCATTGCGCAATCGTATACCGAC -3`

pTRG-EcoRI-for	
dgcZ-GGDEF-pBT-EcoRI-for	5`-GCC**GAATTC**CCTGAATCTTTATTTAATGTTG-3`
dgcZ-GGDEF-pTRG-EcoRI-for	5`-GCC**GAATTC**CCCTGAATCTTTATTTAATGTTG-3`
pdeA-GGDEF-XhoI-rev	5`-GCC**CTCGAG**TTAATTGTTCCAGTGAATTTGCCG-3`
pdeF-GGDEF-XhoI-rev	5`-GCC**CTCGAG**TTATAGTCGATTCATCGCGACTTT-3`
pdeK-GGDEF-XhoI-rev	5`-GCC**CTCGAG**TCAGTCCCAACTGCCATCCGG-3`
pdeO-GGDEF-NotI-for	5`-TTT**GCGGCCGC**ATCTCCCGTGGTGTATCTCATC-3`
pdeO-GGDEF-XhoI-rev	5`-GCC**CTCGAG**TTACTCTTTTACCATTTCGTT-3`
pdeR-PAS-XhoI-rev	5`-GCTA**CTCGAG**GTGATCGATTAAATCCTGC-3`
pdeR-GGDEF-pTRG-EcoRI-for	5`-GCC**GAATTC**AGCGCAATAACCGTGTATTTTTTCG-3`
pdeR-GGDEF-XhoI-rev	5`-GCTA**CTCGAG**CCAGGTGATTTTCGGTTGATAGTGAATA-3`
pdeR-EAL-pTRG-EcoRI-for	5`-GCC**GAATTC**CAGCGTCACGCCGACACAGCAATG-3`
csrD-GGDEF-XhoI-rev	5`-GCC**CTCGAG**TTAACGCCAGCGAACATTACCGCG-3`
pBT-test-for	5`-TCCGTTGTGGGGAAAGTTATC-3`
pBT-test-rev	5`-GGGTAGCCAGCAGCATCC-3`
pTRG-test-for	5`-TGGCTGAACAACTGGAAGCT-3`
pTRG-test-rev	5`-AATCGTCGCCCGCCATAA-3`

II. Primers used for C-terminal 3xFLAG-tagging:

bluF-d-H1P1(FLAG)	5'-TTGAATGGTATCCCTTCCATAGCGTGGCCAGAGAAAAAA<br>GACTACAAAGACCATGACGG-3'
bluF-u-H2P2(FLAG)	5'-ATGATTTACTGAAATCATGTGAAAGAATGTGCTGAAAATTA<br>CCTTAGTTCCTATTCCGAAGTTCC-3'
cdgI-d-H1P1(FLAG)	5'-GAAAAGCGAGGGGGGCAACAAAGTGATTATTCATCATATT<br>GACTACAAAGACCATGACGG-3'
cdgI-u-H2P2(FLAG)	5'-AAAAGCCACGCAAAAAGCATGGCTCTGAATATTTTTGCACTTA<br>CATATGAATATCCTCCTTAG-3'
csrD-d-H1P1(FLAG)	5'-TGATACTAACGTGAAAAAATATTCACAAAGATACTCGGTT<br>GACTACAAAGACCATGACGG-3'
csrD-u-H2P2(FLAG)	5'-CGCATTATTCTACGTGAAAACGGATTAAACGGCAGGTTA<br>CCTTAGTTCCTATTCCGAAGTTCC-3'
dgcC-d-H1P1(FLAG)	5'-AGCAAAGAAAGCCGGACGTAACCGCACCGAAGTGGCGGCC<br>GACTACAAAGACCATGACGG-3'
dgcC-u-H2P2(FLAG)	5'-GAAAAACTCAGCAAATCCTGATGACTTTCGCCGGACGTCA<br>CCTTAGTTCCTATTCCGAAGTTCC-3'
dgcE-d-H1P1(FLAG)	5'-GCTGGATTTGCTGGTGAATAGTAGTTATTTCGCGATTAAC<br>GACTACAAAGACCATGACGG-3'
dgcE-u-H2P2(FLAG)	5'-CGCGTTAGCGTCGCATCAGGCGATGGGGAAGCACGCCTCA<br>CCTTAGTTCCTATTCCGAAGTTCC-3'
dgcF-d-H1P1(FLAG)	5'-CAACCGTACCAGCACTATGCGTTACGGTGAGGAAGTTGTC<br>GACTACAAAGACCATGACGG-3'
dgcF-u-H2P2(FLAG)	5'-CACATTGCATTCAAATCGCGCGTAATGAATAAAGATGTCA<br>CATATGAATATCCTCCTTAG-3'
dgcI-d-H1P1(FLAG)	5'-CAGAAACCCATGCCATTCTCCGCCTGGGATAAAAGTGGAAAATTA<br>GACTACAAAGACCATGACGG-3'
dgcI-u-H2P2(FLAG)	5'-GTACGAACTTATTGATTCTGGACATACGTAAACTACTCTTTTAC<br>CCTTAGTTCCTATTCCGAAGTTCC-3'
dgcJ-d-H1P1(FLAG)	5'-GATGAGCGTTTATATGTCAATAAGCAGAACAAAAACAGCCGTTCA

	GACTACAAAGACCATGACGG-3’
dgcJ-u-H2P2(FLAG)	5’-CATTTCACGCTCCTGAGATTACAAGCAAACAACCACAGAAGGTTA<br>TCACCTTAGTTCCTATTCCGAAGTTCC-3’
dgcM-d-H1P1(FLAG)	5’-TCGGGCGAAAAATGATGGACGCAACCGCGTGCTGGCGGCA<br>GACTACAAAGACCATGACGG-3’
dgcM-u-H2P2(FLAG)	5’-CTAAGCGCAGTCGTTGATCTCGAGACGCATCCGCGGCTTA<br>CCTTAGTTCCTATTCCGAAGTTCC-3’
dgcN-d-H1P1(FLAG)	5’-ATATGTATCAGGCCAAACACCAGCGTGCCGAAAAGCTGGTGAGA<br>GACTACAAAGACCATGACGG-3’
dgcN-u-H2P2(FLAG)	5’-GGGGTGCTACCAGGTGCTTTATCATATCGATATATCCTTGTTA<br>CCTTAGTTCCTATTCCGAAGTTCC-3’
dgcO-d-H1P1(FLAG)	5’-ACGAGGTAGAAACCGTGTTGAACTCTGGAAAGCCAGTCTT<br>GACTACAAAGACCATGACGG-3’
dgcO-u-H2P2(FLAG)	5’-CGGTTAGCTTCATGATTACCTCTGCATCCTGGCGCATCTA<br>CCTTAGTTCCTATTCCGAAGTTCC-3’
dgcP-d-H1P1(FLAG)	5’-ACAGAAAACACCTTTTGTCGCGCATCCAGCGCTACATTCC<br>GACTACAAAGACCATGACGG-3’
dgcP-u-H2P2(FLAG)	5’-CGCATGTTATCACCAATCAAAAGGATGTGAATACGCCTCA<br>CCTTAGTTCCTATTCCGAAGTTCC-3’
dgcQ-d-H1P1(FLAG)	5’-TAAACAGGCTGGGCGTAATCGGGTATTCGCGAGCGATAAC<br>GACTACAAAGACCATGACGG-3’
dgcQ-u-H2P2(FLAG)	5’-GGCGTGAAGGGCTGGACCATTTTTTCTCCGCCCGTTAAGC<br>CCTTAGTTCCTATTCCGAAGTTCC-3’
dgcT-d-H1P1(FLAG)	5’-TAATAAGGTGGTTGTGAGGGATGTGGTGAATTTTTGTGAGTCACCA<br>GACTACAAAGACCATGACGG-3’
dgcT-u-H2P2(FLAG)	5’-GGCCTGTGTCCATATTACGTGGGTAGGATCAAAATGCCGCTTTA<br>CCTTAGTTCCTATTCCGAAGTTCC-3’
dgcZ-d-H1P1(FLAG)	5’-AGCAAACCGGAAGAAATCGCTGCATGTTTATTGACGAACAAAAT<br>GTGGACTACAAAGACCATGACGG-3’
dgcZ-u-H2P2(FLAG)	5’-CATCAGTTTTCTCAATGAATGTTAAACGGAGCTTAAACTCGGTTAAT<br>CCTTAGTTCCTATTCCGAAGTTCC-3’
pdeA-d-H1P1(FLAG)	5’-GCAATATCTGCAAGGGTATTTGATTGGTCGCCCGCAGCCATTAGCT<br>GATGACTACAAAGACCATGACGG-3’
pdeA-u-H2P2(FLAG)	5’-AAGTTAGCGCTTATGGGATAATTCCCCGGTTTTTTACGCCTGTTA<br>CCTTAGTTCCTATTCCGAAGTTCC-3’
pdeB-d-H1P1(FLAG)	5’-GGCATTACCGAAAGAAGATTTCTTACGCTGGGCCGAGCAACATTTG<br>GACTACAAAGACCATGACGG-3’
pdeB-u-H2P2(FLAG)	5’-GGTAGCGTGACGGGCTGTGCGGCGTGCCGTCAGCCCAAAAATTCA<br>CCTTAGTTCCTATTCCGAAGTTCC-3’
pdeC-d-H1P1(FLAG)	5’-GCAATGGATGGAGCAATTACCCGCGCGGGAGTTAACGCGCGGG<br>CAAGACTACAAAGACCATGACGG-3’
pdeC-u-H2P2(FLAG)	5’-CAGTTTGATCGCACTCCCAGCGATTATCGCCACCGCCTGTAATTTTA<br>CCTTAGTTCCTATTCCGAAGTTCC-3’
pdeD-d-H1P1(FLAG)	5’-CCGGCATAATGGACATATCACGCCCATTATGCCGTTACGT<br>GACTACAAAGACCATGACGG-3’
pdeD-u-H2P2(FLAG)	5’-TAAAGAGCAACCGGCGCACGATGAAGATGAGTAAGCGTTA<br>CCTTAGTTCCTATTCCGAAGTTCC-3’
pdeF-d-H1P1(FLAG)	5’-GTTAATTGATACGCTGAATGAAATCGAACCCATTCGCGAAAGTGCC<br>GACTACAAAGACCATGACGG-3’
pdeF-u-H2P2(FLAG)	5’-TACTTTGCGGGCCGACGAGAAATGTCGGCCCGCATTATTCA<br>CCTTAGTTCCTATTCCGAAGTTCC-3’
pdeG-d-H1P1(FLAG)	5’-AATTATCCTTTCTAAACCGAAGGTGAAGGTTGTGGTTGAG<br>GACTACAAAGACCATGACGGTG-3’
pdeG-u-H2P2(FLAG)	5’-TATAATTACACCGCACTATGGCCTTACTGATCAATTTTCA<br>CCTTAGTTCCTATTCCGAAGTTCC-3’
pdeH-d-H1P1(FLAG)	5’-GGCACCGATAGAAACGCTGAATACGGCGGTTCTGGCGCTA<br>GACTACAAAGACCATGACGG -3’
pdeH-u-H2P2(FLAG)	5’-CCTAAAGATAGTCCAGCCAGGCGGAAAATGAGGCAGCTTA<br>CCTTAGTTCCTATTCCGAAGTTCC -3’
pdeI-d-H1P1(FLAG)	5’-CAGAAACCCATGCCATTCTCCGCCTGGGATAAAAGTGGAAAATTA

	GACTACAAAGACCATGACGG-3’
pdeI-u-H2P2(FLAG)	5’-GTACGAACTTATTGATTCTGGACATACGTAAACTACTCTTTTAC CCTTAGTTCCTATTCCGAAGTTCC-3’
pdeK-d-H1P1(FLAG)	5’-CCCTATTGAAATCTTCGAAGAGAGTTACCTGGAAGAAAAG GACTACAAAGACCATGACGG-3’
pdeK-u-H2P2(FLAG)	5’-CAGCACTTTTTAAAGTTTTGTAATCAGTTTGGGGTAGCTA CCTTAGTTCCTATTCCGAAGTTCC-3’
pdeL-d-H1P1(FLAG)	5’-TAATAAATTTATCTCTGAATGGGTAATGAAAGCAGGTGGT GACTACAAAGACCATGACGG-3’
pdeL-u-H2P2(FLAG)	5’-CCAACCTCCCGATGCGCTGTGAATAGCGGTTTACATCA CATATGAATATCCTCCTTAG-3’
pdeN-d-H1P1(FLAG)	5’-TTACCGCTGGACGATTTTGTTCGCTGGCTAAAGAAACCGTATACG GACTACAAAGACCATGACGG-3’
pdeN-u-H2P2(FLAG)	5’-CCGGCATGCTTTCACTATGAATAATAAGGGACGTAAGCACACCTTA CCACTGCGGCCTTAGTTCCTATTCCGAAGTTCC-3’
pdeO-d-H1P1(FLAG)	5’-AATTCCAGGCTGGATGAGCAGCGTGTTACCGCTGAAAATC GACTACAAAGACCATGACGG-3’
pdeO-u-H2P2(FLAG)	5’- TAAATGAAAACCCGCGAGTGCGGGCGAGAGGAATTTGTCA CCTTAGTTCCTATTCCGAAGTTCC-3’
rflP-d-H1P1(FLAG)	5’-GCCTGCCGTTCCCATAAATCAGATAACGACGTTGGTTCAG GACTACAAAGACCATGACGG-3’
rflP-u-H2P2(FLAG)	5’-GTTACTCTCGTCTTAAACACCAGCAAACAGAAGGGTTATCG CCTTAGTTCCTATTCCGAAGTTCC-3’

III. Primers used for cloning *pdeH-3xFLAG* and *pdeL-3xFLAG* into pCAB18:

pdeH-EcoRI-for	5´-GGCCCG**GAATTC**AAGGAGGACTGAGATGATAAGGCAGGTTATCC-3´
Flag-HindIII-rev	5´- GGCGCCG**AAGCTT**CTATTTATCGTCGTCATCTTTG -3´
pdeL-d-(-55)*Xma*I	5’-GGAATC**CCCGGG**TAATCACTCATACTGAAC
pdeL-u-1413	5’-GCACGGGCAGGAGTCAATTCTGGTG-3’

IV. Primers used for cloning the cytoplasmic domain of CdgI on pQE30-Xa:

yeaI-960-forward-SacI	5’-CAG**GAGCTC**CAACTCTCCAGC–3’
yeaI-reverse-XmaI	5’-GAT**CCCGGG**TTAAATATGATGAATAATCACTTTG-3’

V. Primers used for generating chromosomal knockout mutations by one-step inactivation:

pdeL- d-(-552)-H1P1	5´-GAATGGATTCAGTCTTAATGAGTGGGTTTTAAGGGACAGGCATAGGT GTAGGCTGGAGCTGCTTC-3´
pdeL- u-1116-H2P2	5´-CCGATGCGCTGTGAATAGCGGTTTACA**TCA**ACCACCTGCTTTCATCAT ATGAATATCCTCCTTAG-3´
